# Towards robust and replicable sex differences in the intrinsic brain function of autism

**DOI:** 10.1186/s13229-021-00415-z

**Published:** 2021-03-01

**Authors:** Dorothea L. Floris, José O. A. Filho, Meng-Chuan Lai, Steve Giavasis, Marianne Oldehinkel, Maarten Mennes, Tony Charman, Julian Tillmann, Guillaume Dumas, Christine Ecker, Flavio Dell’Acqua, Tobias Banaschewski, Carolin Moessnang, Simon Baron-Cohen, Sarah Durston, Eva Loth, Declan G. M. Murphy, Jan K. Buitelaar, Christian F. Beckmann, Michael P. Milham, Adriana Di Martino

**Affiliations:** 1 Donders Center for Brain, Cognition and Behavior, Radboud University Nijmegen, Nijmegen, The Netherlands; 2 Department for Cognitive Neuroscience, Radboud University Medical Center Nijmegen, Nijmegen, The Netherlands; 3 Autism Center, The Child Mind Institute, 101 E 56 Street, New York City, New York 10026 USA; 4 The Margaret and Wallace McCain Centre for Child, Youth and Family Mental Health, Azrieli Adult Neurodevelopmental Centre, and Campbell Family Mental Health Research Institute, Centre for Addiction and Mental Health, Toronto, Canada; 5 Department of Psychiatry and Autism Research Unit, The Hospital for Sick Children, Toronto, Canada; 6 Department of Psychiatry, Temerty Faculty of Medicine, University of Toronto, Toronto, Canada; 7 Autism Research Centre, Department of Psychiatry, University of Cambridge, Cambridge, UK; 8 Department of Psychiatry, National Taiwan University Hospital and College of Medicine, Taipei, Taiwan; 9 Department of Psychology, Institute of Psychiatry, Psychology, and Neuroscience, King’s College London, London, UK; 10 Human Genetics and Cognitive Functions, Institut Pasteur, UMR3571 CNRS, Université de Paris, Paris, France; 11 Sackler Institute for Translational Neurodevelopment, Institute of Psychiatry, Psychology, and Neuroscience, King’s College London, London, UK; 12 Department of Child and Adolescent Psychiatry, Psychosomatics and Psychotherapy, University Hospital Frankfurt am Main, Goethe University, Frankfurt, Germany; 13 Department of Forensic and Neurodevelopmental Sciences, Institute of Psychiatry, Psychology, and Neuroscience, King’s College London, London, UK; 14 Department of Child and Adolescent Psychiatry, Central Institute of Mental Health, University of Heidelberg, Mannheim, Germany; 15 Department of Psychiatry and Psychotherapy, Central Institute of Mental Health, University of Heidelberg, Mannheim, Germany; 16 Department of Psychiatry, Brain Center Rudolf Magnus, University Medical Center Utrecht, Utrecht, the Netherlands; 17 Karakter Child and Adolescent Psychiatry University Centre, Nijmegen, the Netherlands; 18 Centre for Functional MRI of the Brain, University of Oxford, Oxford, UK; 19 Nathan Kline Institute for Psychiatric Research, Orangeburg, NY USA; 20 Department of Applied Psychology: Health, Development, Enhancement, and Intervention, University of Vienna, Vienna, Austria; 21 CHU Sainte-Justine Research Center, Department of Psychiatry, Université de Montréal, Montreal, QC Canada

**Keywords:** Autism spectrum disorder, Resting-state functional connectivity, Sex differences, Replication, Robustness, Voxel-mirrored homotopic connectivity

## Abstract

**Background:**

Marked sex differences in autism prevalence accentuate the need to understand the role of biological sex-related factors in autism. Efforts to unravel sex differences in the brain organization of autism have, however, been challenged by the limited availability of female data.

**Methods:**

We addressed this gap by using a large sample of males and females with autism and neurotypical (NT) control individuals (ABIDE; Autism: 362 males, 82 females; NT: 409 males, 166 females; 7–18 years). Discovery analyses examined main effects of diagnosis, sex and their interaction across five resting-state fMRI (R-fMRI) metrics (voxel-level Z > 3.1, cluster-level *P* < 0.01, gaussian random field corrected). Secondary analyses assessed the robustness of the results to different pre-processing approaches and their replicability in two independent samples: the EU-AIMS Longitudinal European Autism Project (LEAP) and the Gender Explorations of Neurogenetics and Development to Advance Autism Research.

**Results:**

Discovery analyses in ABIDE revealed significant main effects of diagnosis and sex across the intrinsic functional connectivity of the posterior cingulate cortex, regional homogeneity and voxel-mirrored homotopic connectivity (VMHC) in several cortical regions, largely converging in the default network midline. Sex-by-diagnosis interactions were confined to the dorsolateral occipital cortex, with reduced VMHC in females with autism. All findings were robust to different pre-processing steps. Replicability in independent samples varied by R-fMRI measures and effects with the targeted sex-by-diagnosis interaction being replicated in the larger of the two replication samples—EU-AIMS LEAP.

**Limitations:**

Given the lack of a priori harmonization among the discovery and replication datasets available to date, sample-related variation remained and may have affected replicability.

**Conclusions:**

Atypical cross-hemispheric interactions are neurobiologically relevant to autism. They likely result from the combination of sex-dependent and sex-independent factors with a differential effect across functional cortical networks. Systematic assessments of the factors contributing to replicability are needed and necessitate coordinated large-scale data collection across studies.

## Background

Autism spectrum disorder (autism) is characterized by a marked male preponderance in prevalence with three times more males being diagnosed than females [1]. This pronounced sex-differential prevalence implies that sex-related biological factors are likely implicated in the neurobiology of autism. However, little is known about the differential underlying neural expressions in males and females with autism. Such knowledge could widen our understanding of potential underlying mechanisms of autism and related neurodevelopmental conditions [2].

This has motivated research into the impact of biological sex on brain organization in autism [2–5]. With the widely accepted view that the neurobiology of autism involves differences in large-scale brain networks [6, 7], resting-state functional magnetic resonance imaging (R-fMRI) has proven to be a valuable complementary tool for investigating atypicalities in intrinsic functional connectivity (iFC). While the exact nature of the intrinsic brain organization in autism remains to be established [6], research on the impact of biological sex differences in autism is just beginning to emerge.

Several R-fMRI studies have focused on autism-related sex differences in iFC [2, 8–18]. They vary on the extent of the functional networks and intrinsic properties examined. Most of them examined the strength of iFC between one or more regions/networks selected a priori [8–12, 14, 15], or via data-driven analyses [18]. A few others investigated either local or homotopic iFC across the whole brain [2, 13, 17]. Across these different efforts, the pattern of findings have also been mixed; some studies supported the predictions from the ‘extreme male brain’ theory [12, 13], whereas others supported the predictions from the ‘gender-incoherence’ theory [8, 11, 14, 15, 18]. The ‘extreme male brain’ model predicts that brain characteristics in males and females with autism will resemble those in neurotypical males (i.e., shifts towards maleness in both sexes [19]). R-fMRI results consistent with a shift towards maleness in autism were reported in both Ypma et al. [12] and Kozhemiako et al. [13, 17]. The ‘gender incoherence’ model predicts that brain characteristics in females with autism resemble those of neurotypical males, whereas brain characteristics in males with autism resemble those of neurotypical females (i.e., androgynous patterns in the sexes [20]). The ‘gender incoherence’ model has been supported by findings from prior R-fMRI studies [8, 11, 14], where the results largely revealed hyper-connectivity in females with autism similar to neurotypical (NT) males and hypo-connectivity in males with autism similar to NT females. Such seemingly inconsistent findings of sex-related differences were in part addressed by Floris et al. [2] who showed that, at least in males with autism, distinct patterns of atypical sex-differentiation coexist, and vary as a function of the neural networks involved. However, the intrinsic brain organization in females with autism has remained largely unclear and the scarce availability of female datasets in most studies may have contributed to the variability in findings in males and females [21, 22].

Accordingly, to explore sex-related atypicalities in autism relative to NT controls, we used, as discovery sample, large R-fMRI datasets of both males and females of autism and NT selected from the Autism Brain Imaging Data Sharing Exchange (ABIDE) [22, 23]. By aggregating neuroimaging datasets from multiple sources, this data sharing initiative has begun to provide a means to address the challenge of underrepresentation of female datasets in autism research. Examining both sexes in both autism and controls allows to directly capture not only sex differences that are common across individuals (i.e., regardless of their diagnosis [main effect of sex]), but also those that are specific to autism and point towards atypical autism-specific sex differential patterns (i.e., sex-by-diagnosis interaction effects) [4]. To do so, given prior inconsistencies in the literature and the limited insights into the brain organization of females with autism, we used a discovery science approach. Unlike most prior work that focused on specific networks or circuits selected a priori*,* we investigated the whole-brain across multiple R-fMRI metrics. We selected R-fMRI metrics capturing unique aspects of the intrinsic brain organization during typical development [24, 25] and, most germane to this study, being reported to be involved in typical sex differences and be affected by autism. They comprised: (1) posterior cingulate cortex (PCC)-iFC—e.g., [2, 12, 23, 26–32]; (2) voxel-mirrored homotopic connectivity (VMHC) [33]—e.g., [13, 32–35]; (3) regional homogeneity (ReHo) [36]—e.g., [17, 32, 37, 38]; (4) network degree centrality (DC) [39]—e.g., [32, 39–41]; and (5) fractional amplitude of low frequency fluctuations (fALFF) [23, 42]—e.g., [23, 32, 43].

Beside the role of small female samples, prior inconsistencies in autism-related sex differences in R-fMRI can be due to other factors that impact reproducibility. For example, while there are growing concerns on the role of pre-processing strategies [44, 45], a recent study showed that their impact on autism-related mean group-differences is minimal [46]. Additionally, while several studies have reported some degree of consistency on R-fMRI findings across either independent, or partially overlapping, samples [41, 47–49], results from other studies have raised concerns on the replicability of group-mean diagnostic effects [46, 50]. However, none of these studies have explicitly examined robustness and replicability of sex-by-diagnosis interaction effects, which account for a potentially relevant source of variability in autism—biological sex. Thus, we conducted secondary analyses to assess the extent to which the pattern of findings obtained in our discovery analyses were also observed (a) after applying different nuisance pre-processing steps that have been previously validated, though used inconsistently in the autism literature [46], and (b) across two independent, multisite R-fMRI datasets: the EU-AIMS Longitudinal European Autism Project (LEAP) [51, 52] and the Gender Explorations of Neurogenetics and Development to Advance Autism Research (GENDAAR) dataset [53]—i.e., *robustness* and *replicability*.

## Methods

### Discovery sample: ABIDE I and II

For discovery analyses, we examined the R-fMRI dataset with one of the largest number of females and males in both the autism and the NT groups available to date, selected from the Autism Brain Imaging Data Exchange (ABIDE) repositories ABIDE I and II [22, 23]. The final ABIDE I and II dataset of *N* = 1019 included *N* = 82 females with autism, *N* = 362 males with autism, *N* = 166 neurotypical females (NT F), and *N* = 409 neurotypical males (NT M), aggregated across 13 sites. Specific selection criteria are described in Supplementary Methods in the Additional file 1 and depicted as a figure in the Additional file 1: Figure S1. Briefly, we selected cases between 7 and 18 years of age (the ages most represented across ABIDE sites), with MRI data successfully completing brain image co-registration and transformation to standard space, with FIQ between 70 and 148 and with mean framewise displacement (mFD) [54] within three times the interquartile range (IQR) + the third quartile (Q3) of the sample (i.e., mFD 0.39 mm). Further steps included matching for mean age *across* groups, as well as for mFD and IQ *within* diagnostic groups. This latter step limited the number of exclusions while keeping average group motion low (mFD < 0.2 mm) and sampling biases that may result when matching neurodevelopmental conditions to NT around intrinsic features such as IQ [55, 56]. At each step, any sites with less than three individual datasets per diagnostic/sex groups were excluded. Demographics and characteristics of this sample are summarized in Table 1 and in Supplementary Methods in the Additional file 1.

**Table 1:** Characterization of sample merged across ABIDE I and II Cluster masks are overlaid on inflated brain maps generated by BrainNet Viewer

ABIDE I + II	Sites^a^	ASD M (*N* = 362)	ASD F (*N* = 82)	NT M (*N* = 409)	NT F (*N* = 166)	Statistics	Post-hoc
	*N*	Mean (SD) [Range]	Mean (SD) [Range]	Mean (SD) [Range]	Mean (SD) [Range]		
Age	13	11.8 (2.6) [7–17.9]	11.7 (2.7) [7–18]	11.8 (2.6) [7.1–18.2]	11.4 (2.3) [7.8–17.4]	*F*_(3)_ = 1.18 *p* = 0.32	
Full-Scale IQ^b^	13	106 (16.6) [72–148]	104 (16.3) [73–147]	112 (12.7) [73–148]	114 (12.7) [80–144]	*F*_(3)_ = 19.24 *p* < 0.001	(ASD M = ASD F) < (NT M = NT F)
Verbal IQ^c^	12	107 (17.9) [57–180]	105 (17.3) [62–145]	114 (13.5) [73–147]	114 (14.4) [83–146]	*F*_(3)_ = 16.78 *p* < 0.001	(ASD M = ASD F) < (NT M = NT F)
Performance IQ^d^	12	106 (17.0) [59–157]	104 (17.1) [67–148]	109 (14.2) [62–147]	109 (13.2) [79–145]	*F*_(3)_ = 3.1 *p* = 0.03	(ASD M = ASD F) < (NT M = NT F)
Mean FD	13	0.11 (0.07) [0.02–0.39]	0.13 (0.09) [0.02–0.39]	0.09 (0.06) [0.02–0.39]	0.09 (0.06) [0.02–0.38]	*H*_(3)_ = 29.6 *p* < 0.001	(ASD M = ASD F) < (NT M = NT F)
*ADI-R*							
Social^e^	11	19.7 (5.2) [4–30]	19.6 (5.5) [7–30]	–	–	*t*_(93)_ = 0.14 *p* = 0.89	
Communication^f^	11	15.6 (4.5) [2–25]	15.2 (5.0) [4–24]	–	–	*t*_(92)_ = 0.61 *p* = 0.54	
RRB^f^	11	6.0 (2.4) [0–13]	5.8 (2.5) [0–12]	–	–	*t*_(96)_ = 0.51 *p* = 0.61	
*ADOS-2*							
Social-Affect^g^	11	9.1 (3.7) [1–20]	8.7 (3.2) [4–18]	–	–	*t*_(87)_ = 0.97 *p* = 0.33	
RRB^h^	11	3.2 (1.8) [0–8]	2.8 (1.5) [0–5]	–	–	*t*_(93)_ = 1.79 *p* = 0.08	
CSS total^i^	11	6.9 (2.1) [1–10]	6.8 (1.8) [2–10]	–	–	*t*_(93)_ = 0.11 *p* = 0.32	
		*N*	*N*			Statistics	Post-hoc
Comorbidity	5	99^j^	25^k^	–	–	*χ*^2^_(1)_ = 0.2 *p* = 0.66	
Psychoactive medication	10	112	26	–	–	*χ*^2^_(1)_ < 0.01 *p* = 0.99	

*ABIDE* Autism Brain Imaging data exchange, *ADI-R* Autism Diagnostic Interview-Revised, *ADOS-2* Autism Diagnostic Observation Schedule-2, *ASD* Autism Spectrum Disorder, *CSS* Calibrated Severity Score, *F* females, *IQ* intellectual quotient, *M* males, *Mean FD* mean framewise displacement [54]; *NT* neurotypical, *RRB* restricted repetitive behaviors

^a^ABIDE I data collections: KKI, Leuven2, NYU, OHSU, Pitt, SDSU, Stanford, UCLA1, UM1, and Yale. ABIDE II data collections: ABIDEII-GU1, ABIDEII-KKI1, ABIDEII-KKI2, ABIDEII-NYU1, ABIDEII-OHSU1, ABIDEII-SDSU1, ABIDEII-UCD1 and ABIDEII-UCLA1. KKI and ABIDEII-KKI1, NYU and ABIDEII-NYU1, SDSU and ABIDEII-SDSU1, OHSU and ABIDEII-OHSU1 and UCLA1 and ABIDEII-UCLA1 were merged into one site across ABIDE I and ABIDE II collections

^b^FIQ was available for 362 males with ASD (2 missing from UM1, ABIDEII-SDSU1), 81 females with ASD (1 missing from ABIDEII-GU1), 407 neurotypical males (NT M) [3 missing from ABIDEII-GU1 (*N* = 1), UM1 (*N* = 2)] and all 166 NT females (NT F)

^c^VIQ was available for 315 males with ASD (47 missing; KKI (*N* = 14), ABIDEII-OHSU1 (*N* = 22), OHSU (*N* = 9), ABIDEII-SDSU1 (*N* = 1); ABIDEII-UCLA1 (*N* = 1), 70 females with ASD [12 missing, ABIDEII-GU1 (*N* = 1), KKI (*N* = 4), ABIDEII-OHSU1 (*N* = 7)], 351 NT M [59 missing; ABIDEII-GU1 (*N* = 1), KKI (*N* = 23), OHSU (*N* = 15), ABIDEII-OHSU1 (*N* = 20)], and 139 NT F [27 missing; KKI (*N* = 8), ABIDEII-OHSU1 (*N* = 19)]

^d^PIQ was available for 306 males with ASD [56 missing; ABIDEII-GU1 (*N* = 9), KKI (*N* = 14), OHSU (*N* = 9), ABIDEII-OHSU1 (*N* = 22), ABIDEII-UCLA1 (*N* = 1), UM1, (*N* = 1)], 67 females with ASD [15 missing; ABIDEII-GU1 (*N* = 4), KKI (*N* = 4), ABIDEII-OHSU1 (*N* = 7)], 349 NT M [61 missing; ABIDEII-GU1 (*N* = 1), KKI (*N* = 23), OHSU (*N* = 15), ABIDEII-OHSU1 (*N* = 20), UM1, (*N* = 2)], 139 NT F [27 missing; KKI (*N* = 8), ABIDEII-OHSU1 (*N* = 19)]

^e^ADI-R Social scores were available for 317 males with ASD [45 missing; ABIDEII-GU1 (*N* = 1), Leuven2 (*N* = 10), NYU (*N* = 3), ABIDEII-NYU1 (*N* = 1), SDSU (*N* = 2), ABIDEII-UCD1 (*N* = 11), ABIDEII-UCLA1 (*N* = 14), UM1, (*N* = 2), Yale (*N* = 3)] and 68 females with ASD [14 missing; ABIDEII-GU1 (*N* = 1), ABIDEII-KKI1 (*N* = 2), Leuven2 (*N* = 3), NYU (*N* = 1), Pitt (*N* = 1), ABIDEII-UCD1 (*N* = 3), ABIDEII-UCLA1 (*N* = 1), Yale (*N* = 2)]

^f^ADI-R Communication and RRB scores were available for 318 males with ASD [45 missing; ABIDEII-GU1 (*N* = 1), Leuven2 (*N* = 10), NYU (*N* = 2), ABIDEII-NYU1 (*N* = 1), SDSU (*N* = 2), ABIDEII-UCD1 (*N* = 11), ABIDEII-UCLA1 (*N* = 14), UM1, (*N* = 2), Yale (*N* = 3)] and 68 females with ASD [14 missing; ABIDEII-GU1 (*N* = 1), ABIDEII-KKI1 (*N* = 2), Leuven2 (*N* = 3), NYU (*N* = 1), Pitt (*N* = 1), ABIDEII-UCD1 (*N* = 3), ABIDEII-UCLA1 (*N* = 1), Yale (*N* = 2)]

^g^ADOS-Gotham Social-Affect was available for 261 males with ASD [101 missing; ABIDEII-GU1 (*N* = 27), ABIDEII-KKI1 (*N* = 13), Leuven2 (*N* = 10), NYU (*N* = 7), OHSU (*N* = 11), Pitt (*N* = 8), SDSU (*N* = 8), Stanford (*N* = 6), ABIDEII-UCLA1 (*N* = 5), UM1 (*N* = 6), Yale (*N* = 1)] and 55 females with ASD [27 missing; ABIDEII-GU1 (*N* = 6), ABIDEII-KKI1 (*N* = 7), Leuven2 (*N* = 3), ABIDEII-OHSU1 (*N* = 1), Pitt (*N* = 4), Stanford (*N* = 1), ABIDEII-UCD1 (*N* = 1), UCLA1 (*N* = 1), UM1 (*N* = 3)]

^h^ADOS-Gotham RRB was available for 264 males with ASD [98 missing; ABIDEII-GU1 (*N* = 27), ABIDEII-KKI1 (*N* = 13), Leuven2 (*N* = 10), NYU (*N* = 7), OHSU (*N* = 11), Pitt (*N* = 8), SDSU (*N* = 8), Stanford (*N* = 3), ABIDEII-UCLA1 (*N* = 5), UM1 (*N* = 6), Yale (*N* = 1)] and 56 females with ASD [26 missing; ABIDEII-GU1 (*N* = 6), ABIDEII-KKI1 (*N* = 7), Leuven2 (*N* = 3), ABIDEII-OHSU1 (*N* = 1), Pitt (*N* = 4), ABIDEII-UCD1 (*N* = 1), UCLA1 (*N* = 1), UM1 (*N* = 3)]

^i^ADOS-Gotham calibrated severity scores [65] were available for 347 males with ASD (15 missing) and 77 females with ASD (5 missing)

^j^Attention Deficit Hyperactivity Disorder (ADHD; *N* = 63); anxiety disorder (*N* = 22); Oppositional Defiant Disorder (ODD; *N* = 17); mood disorder (*N* = 11); Tourettes/Tics (*N* = 6); Obsessive–Compulsive Disorder (OCD; *N* = 6); enuresis (*N* = 8); encopresis (*N* = 4); developmental articulation disorder (*N* = 1); developmental dyslexia (*N* = 1); sensory integration disorder (*N* = 1)

^k^ADHD (*N* = 17); anxiety disorder (*N* = 7); ODD (*N* = 10); mood disorder (*N* = 2); OCD (*N* = 2); enuresis (*N* = 2); encopresis (*N* = 1). The three group means were compared with ANOVA tests (or Kruskal–Wallis test in the case of non-parametric mean FD) followed by post-hoc pairwise *t* test comparisons (or Mann–Whitney *U* tests in the case of non-parametric mean FD) when statistically significant (significance cut-off set at *p* < 0.05)

### Discovery analysis pre-processing pipeline

We examined five whole-brain R-fMRI metrics previously reported to reflect typical sex differences and found to be atypical in autism, including (1) PCC-iFC, (2) VMHC, (3) ReHo, (4) DC and (5) fALFF (see Additional file 1: Supplementary Methods). R-fMRI image pre-processing steps included: slice time correction, 24 motion parameters regression [57], component-based noise reduction (CompCor) [58], removal of linear and quadratic trends, and band-pass filtering (0.01–0.1 Hz, for all metrics but fALFF). Functional-to-anatomical co-registration was achieved by Boundary Based Registration (BBR) using FSL FLIRT [59]. Linear and nonlinear spatial normalization of functional echo planar images (EPIs) to Montreal Neurological Institute 152 (MNI152) stereotactic space (2 mm^3^ isotropic) was done using ANTS registration (Advanced Neuroimaging Tools) [60]. Computation of voxel-mirrored homotopic connectivity (VMHC) followed registration to a symmetric template. All R-fMRI derivatives were smoothed by a 6 mm FWHM Gaussian kernel. To account for site and collection time variability across each of the data collections in ABIDE I and II data repositories, site effects were removed using the ComBat function available in python [61] (https://github.com/brentp/combat.py). This approach has been shown to effectively account for scanner-related variance in multi-site R-fMRI data [61]. For further details see Additional file 1: Supplementary Methods.

### Discovery group-level analyses

Statistical Z-maps were generated within study-specific functional volume masks including all voxels in MNI space present across all subjects. Main effects of diagnosis and sex along with their interaction were explored by fitting a general linear model (GLM) including diagnosis or/and sex as the regressors of interest respectively, and age and mean framewise displacement (mFD) [54] as nuisance covariates. In primary analyses, we did not include FIQ as a covariate as this is thought to be suboptimal when comparing groups selected from populations carrying intrinsic IQ differences such as autism and NT [56]. Nevertheless, to provide an indication as to whether IQ may affect primary findings, in supplementary analyses FIQ was also included as an additional nuisance regressor. We applied gaussian random field theory correction based on strict voxel-level threshold of *Z* > 3.1 as recommended by [62] and cluster level *P* < 0.01, given the assessment of five R-fMRI metrics in the same study (i.e., *P* 0.05/5 R-MRI metrics = 0.01).

### Functional relevance of sex differences in autism

Post-hoc analyses were conducted to functionally characterize the sex-by-diagnosis interaction result(s). First, to explore the cognitive domains implicated in the cluster(s), we quantified the percentage of its overlap with 12 cognitive ontology maps [63] thresholded at *P* = 1e−5. We labelled these components based on the top five tasks each component recruits [2]. Second, we used the Neurosynth Image Decoder (http://neurosynth.org/decode/) [64] to visualize the terms most strongly associated with the significant cluster. After excluding anatomical (e.g., occipital) and redundant terms (synonyms [e.g., saccades and eye movements], plurals [e.g., object and objects] or noun/adjective/adverb equivalents [e.g., vision and visual]), we visualized the top 27 terms showing correlations with the cluster map between *r* = 0.64 and *r* = 0.10. Third, to explore potential clinical relevance of the significant cluster, we explored brain-behavior relationships as a function of sex within the autism group. Specifically, we ran a GLM examining the interaction between biological sex and available ADOS calibrated severity total score (CSS) [65], as well as social-affect (SA) and restricted, repetitive behavior (RRB) subscores (see Additional file 1: Supplementary Methods) with the dependent variable(s) being the R-fMRI metric(s) extracted from the cluster mask(s) showing a statistically significant sex-by-diagnosis effect(s).

### Robustness and replicability

#### Robustness

We assessed whether patterns of results from the discovery analyses were observable with two other nuisance regression analytical pipelines that include commonly used data preprocessing steps. One pipeline included global signal regression (GSR) [66] which has often been used in autism studies; the other included Independent Component Analysis-Automatic Removal of Motion Artifacts (ICA-AROMA) [67] which is a relatively novel but increasingly utilized approach [46]. Given the scope of the present study, unlike prior work focusing on a wide range of individual preprocessing pipelines [46], we selected GSR and ICA-AROMA as examples of previously validated approaches thought to have impact on motion and physiological noise [45]. To assess robustness of the results observed in discovery analyses, following the voxel-level GLM, we extracted means from the masks corresponding to the same clusters that showed significant effects. These values were averaged across all the voxels in the cluster mask for a given R-fMRI metric. We used them to implement a full regression model including the predictors of interest (sex, diagnosis and their interaction), as well as age and mFD as nuisance regressors and compute effect sizes as partial eta squared (*η*_*p*_^2^) and their confidence intervals using the R-package ‘effectsize’. For visualization purposes we also used regressions (including sex, diagnosis, sex-by-diagnosis interaction, age, and mFD) to obtain the residuals of these mask-averaged values.

#### Replicability

Similarly, we assessed whether the group patterns observed in significant clusters identified in discovery analyses were observed in two relatively large-scale, independent datasets selected from (a) the EU-AIMS Longitudinal European Autism Project (LEAP), a large multi-site European initiative aimed at identifying biomarkers in autism [51, 52] and (b) the Gender Explorations of Neurogenetics and Development to Advance Autism Research (GENDAAR) dataset collected by the GENDAAR consortium and shared in the National Database for Autism Research [53]. For details on autism and NT inclusion and exclusion criteria for these samples, as well as our selection process, see Additional file 1: Supplementary Methods [52, 53]. The resulting EU-AIMS LEAP (*N* = 309) R-fMRI datasets comprised *N* = 133 males and *N* = 43 females with autism as well as *N* = 85 NT males, and *N* = 48 NT females (see Additional file 1: Table S1); resulting GENDAAR (*N* = 196) R-fMRI datasets comprised *N* = 43 males and *N* = 44 females with autism, as well as *N* = 56 NT males and *N* = 53 NT females (see Additional file 1: Table S2). For a comparison of demographic and clinical information between ABIDE, EU-AIMS LEAP and GENDAAR, see Table S3, S4 and S5 in the Additional file 1. After applying the same ComBat and pre-processing pipeline as used in the ABIDE-based discovery analyses, we extracted each of the R-fMRI metrics means from the Z-maps. As for robustness, we extracted values for each R-fMRI metric from the masks corresponding to the clusters showing statistically significant effects in discovery analyses and computed the corresponding effect sizes and residuals using the same methods described above.

For both robustness and replicability, discovery findings were determined to be robust and/or replicable (R+) based on two criteria: (1) the group mean difference(s) observed were in the same direction as those identified in the findings from discovery analyses [68] and (2) their effects were not negligible as defined by partial eta squared (i.e,.*η*_*p*_^2^ ≥ 0.01 any small, medium or large effects were R+) which is also consistent with prior work [41]. Finally, for consistency across analyses we also computed cluster-level effect sizes of the discovery findings using the same approach described above.

## Results

### Discovery analyses: ABIDE

#### Main effects of diagnosis

**Fig. 1:**
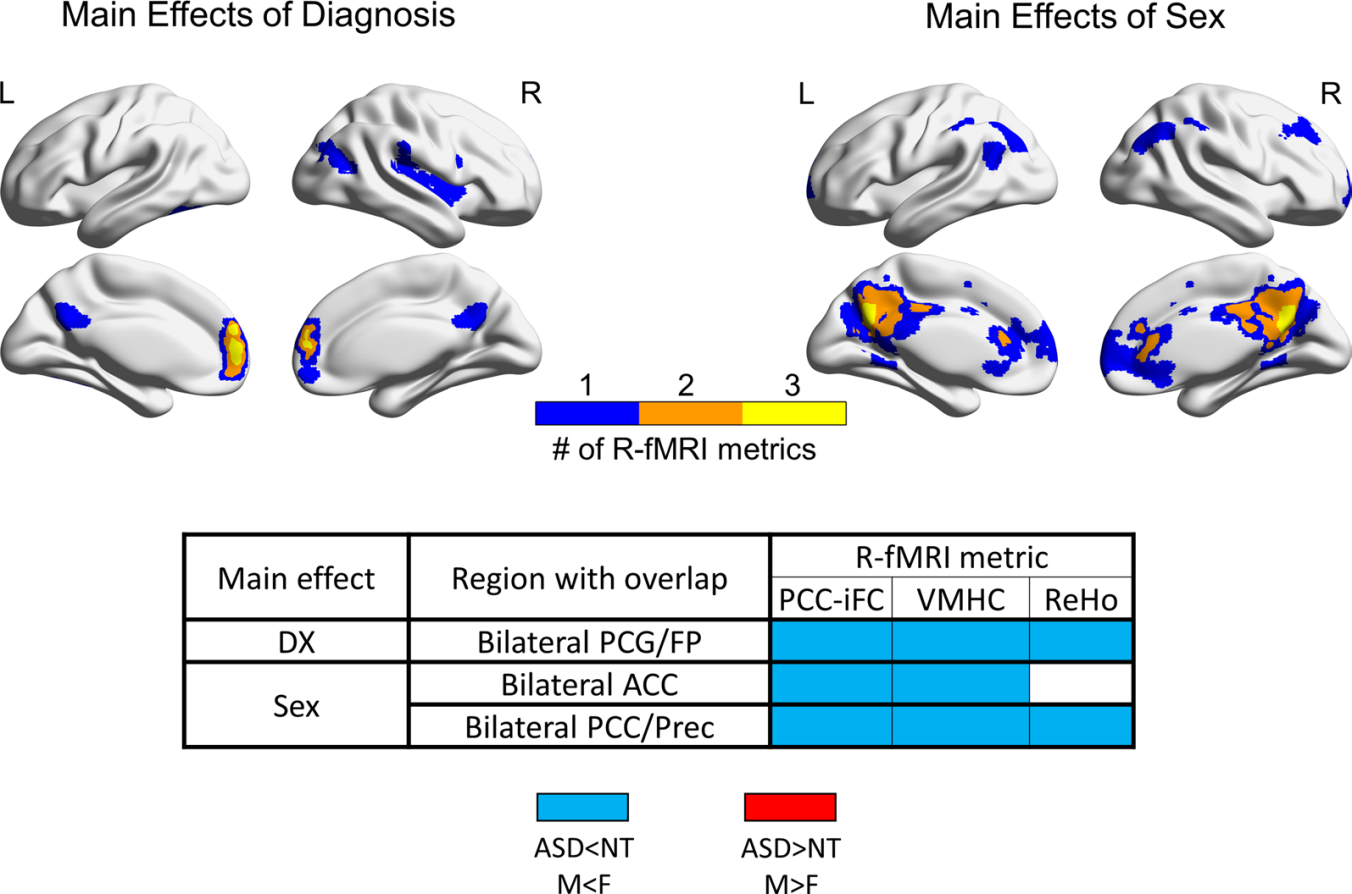
Overlap across R-fMRI metrics for main effects of diagnosis and sex. Upper panel: the surface inflated maps depict the extent of overlap across clusters showing significant main effects of diagnosis (left) and sex (right) across any of three resting state fMRI (R-fMRI) metrics showing statistically significant effects (*Z* > 3.1, *P* < 0.01). Purple clusters represent areas of significant group differences emerging for only one of any of the three R-fMRI measures, orange and yellow clusters indicate measures with overlap among 2 and 3 R-fMRI measures (see Additional file 1: Figure S2 for statistical maps of main effects for each R-fMRI metric). Cluster masks are overlaid on inflated brain maps generated by BrainNet Viewer. Lower panel: For each of the yellow and orange clusters in panel A, the table lists the cluster’s anatomical label based on the Harvard Oxford atlas, the specific R-fMRI metrics involved, and the group difference direction (ASD < NT or M < F in blue, ASD > NT or M > F in red). *L* left hemisphere, *R* right hemisphere, *PCG/FP* paracingulate cortex/frontal pole, *ACC* anterior cingulate cortex, *PCC/Prec* posterior cingulate cortex/precuneus, *ASD* autism spectrum disorder, *NT* neurotypical, *M* males, *F* females

Analyses revealed a total of seven clusters showing a significant effect of diagnosis (voxel-level *Z* > 3.1; cluster-level *P* < 0.01, corrected) for three of the five R-fMRI metrics: PCC-iFC (three clusters), VMHC (two clusters) and ReHo (two clusters); Fig. 1 and Additional file 1: Figure S2. These were mainly evident in anterior and posterior regions of the default network (DN) across at least two or all three R-fMRI metrics. Autism-related hypo-connectivity was present for: (a) PCC-iFC, VMHC and ReHo within bilateral paracingulate cortex and frontal pole, (b) VMHC and ReHo in the bilateral PCC and precuneus, and (c) ReHo in right insula and central operculum (Fig. 1, Additional file 1: Figure S2 and Table S6). Autism-related hyper-connectivity was only evident for PCC-iFC with left superior lateral occipital cortex, temporal occipital fusiform cortex and occipital fusiform gyrus (Additional file 1: Figure S2). These results remained essentially unchanged when additionally controlling for FIQ (Additional file 1: Figure S3). Further, to verify that these findings were not driven by particular acquisition site(s), post-hoc analyses computed group means for diagnostic subgroups for the R-fMRI metrics extracted at the cluster-level masks excluding one out of the 13 ABIDE sites at a time. The pattern of results was essentially unchanged (Additional file 2: Figure S5a).

#### Main effects of sex

Analyses revealed clusters showing statistically significant main sex differences (voxel-level *Z* > 3.1; cluster-level *P* < 0.01, corrected), again for three R-fMRI metrics out of five in a total of 10 clusters: PCC-iFC (five clusters), VMHC (three clusters), and ReHo (two clusters). Findings involved lateral and medial portions of the DN with bilateral PCC and precuneus showing the highest overlap (Fig. 1 and Additional file 1: Figure S2). Specifically, regardless of diagnosis, relative to females, males showed decreased PCC-iFC with paracingulate cortex and frontal pole, right middle frontal gyrus, bilateral superior lateral occipital cortex and bilateral PCC and precuneus. Males also showed decreased VMHC and ReHo localized in PCC and precuneus. Decreased ReHo was also evident in the left angular gyrus and lateral occipital cortex in females relative to males (Fig. 1, Additional file 1: Figure S2 and Table S6). These results remained essentially unchanged when additionally controlling for FIQ (Additional file 1: Figure S3). Post-hoc analyses assessing the consistency of these findings across sites, as described above, revealed a similar pattern of results (Additional file 3: Figure S5b).

#### Sex-by-diagnosis interaction effect

**Fig. 2:**
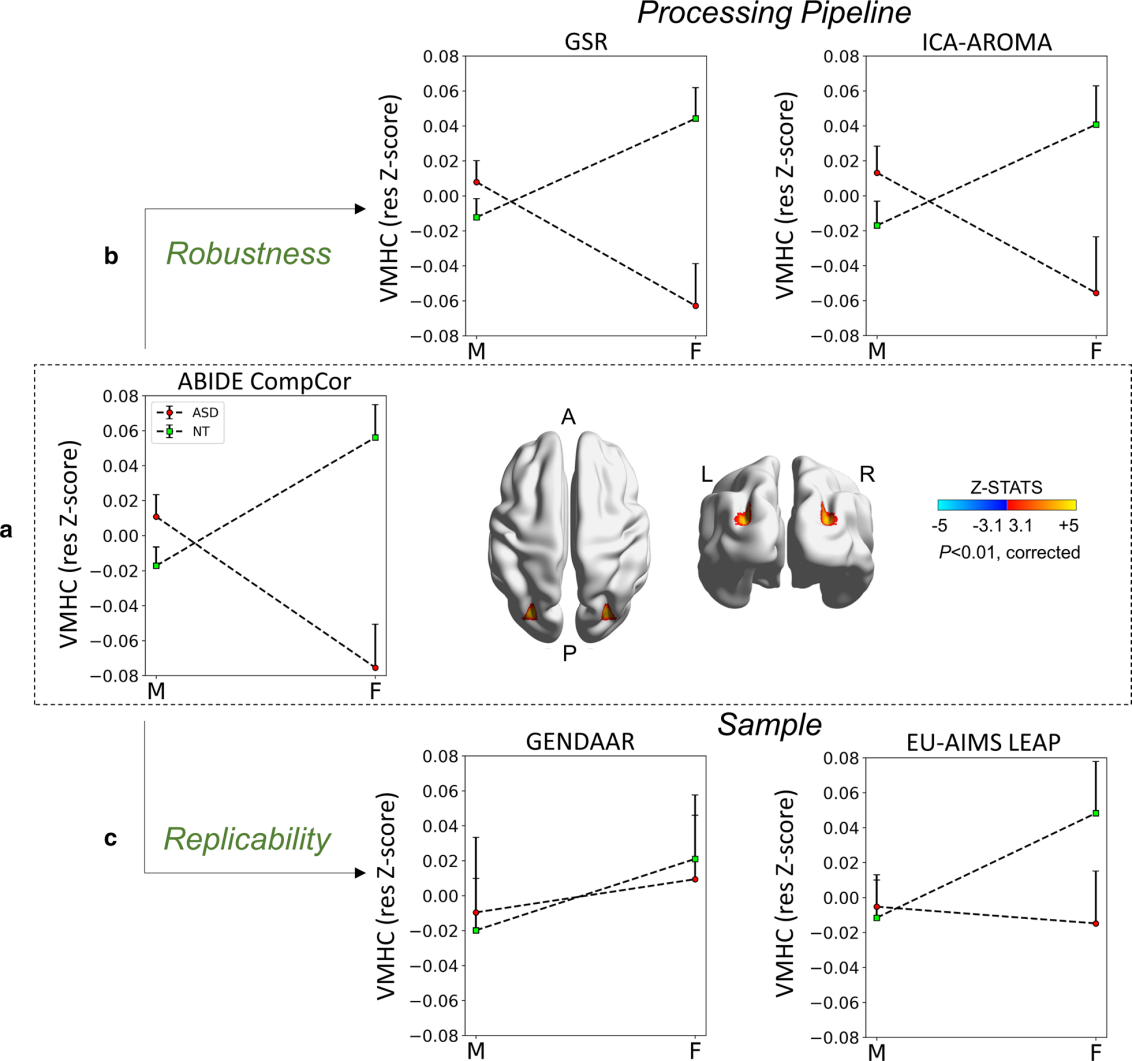
Sex-by-diagnosis interaction effect, its robustness and replicability. (a) On the right, surface maps show the cluster with a significant (*Z* > 3.1, *P* < 0.01) sex-by-diagnosis interaction for voxel-mirrored homotopic connectivity (VMHC) resulting from discovery analyses in the ABIDE sample using the component-based noise reduction (CompCor) pipeline. The statistical Z maps are overlaid on inflated brain maps generated by BrainNet Viewer. (b) The upper panels show the pattern of VMHC group means in males and females by each diagnostic group (ASD and NT) extracted from the same cluster in data pre-processed following two alternative denoising pipelines, Global Signal Regression (GSR, left) and Independent Component Analysis-Automatic Removal of Motion Artifacts (ICA-AROMA, right). Results show a pattern similar to the those observed in discovery analyses with small to moderate effect sizes (*η*_*p*_^2^ range = 0.01–0.07). (c) The lower graph shows replicability in two independent samples: the Gender Explorations of Neurogenetics and Development to Advance Autism Research (GENDAAR) and the EU-AIMS Longitudinal European Autism Project (LEAP). The pattern of results was replicable in the EU-AIMS LEAP (*N* = 309) with a small effect size (*η*_*p*_^2^ = 0.01) and had a negligible effect size in GENDAAR (*N* = 196; *η*_*p*_^2^ < 0.01). For all graphs VMHC data are shown as residuals obtained after regressing out mean framewise displacement and age effects. *L* left, *R* right, *A* anterior, *P* posterior

Statistically corrected voxel-wise analyses (voxel-level *Z* > 3.1; cluster-level *P* < 0.01) revealed one cluster of significant sex-by-diagnosis interaction only for VMHC which was localized in the dorsolateral occipital cortex (Fig. 2a and Additional file 1: Table S6). Post-hoc cluster-level group means showed that NT females had higher VMHC than the three other groups, whereas autism females had lower VMHC than the three other groups (Fig. 2a). Similar to the main effects, results remained essentially unchanged when additionally controlling for FIQ (Additional file 1: Figure S3). Analyses assessing the consistency of these findings across sites, as described above, showed a similar pattern of results (Additional file 1: Figure S4).

### Functional relevance of autism-related sex differences

**Fig. 3:**
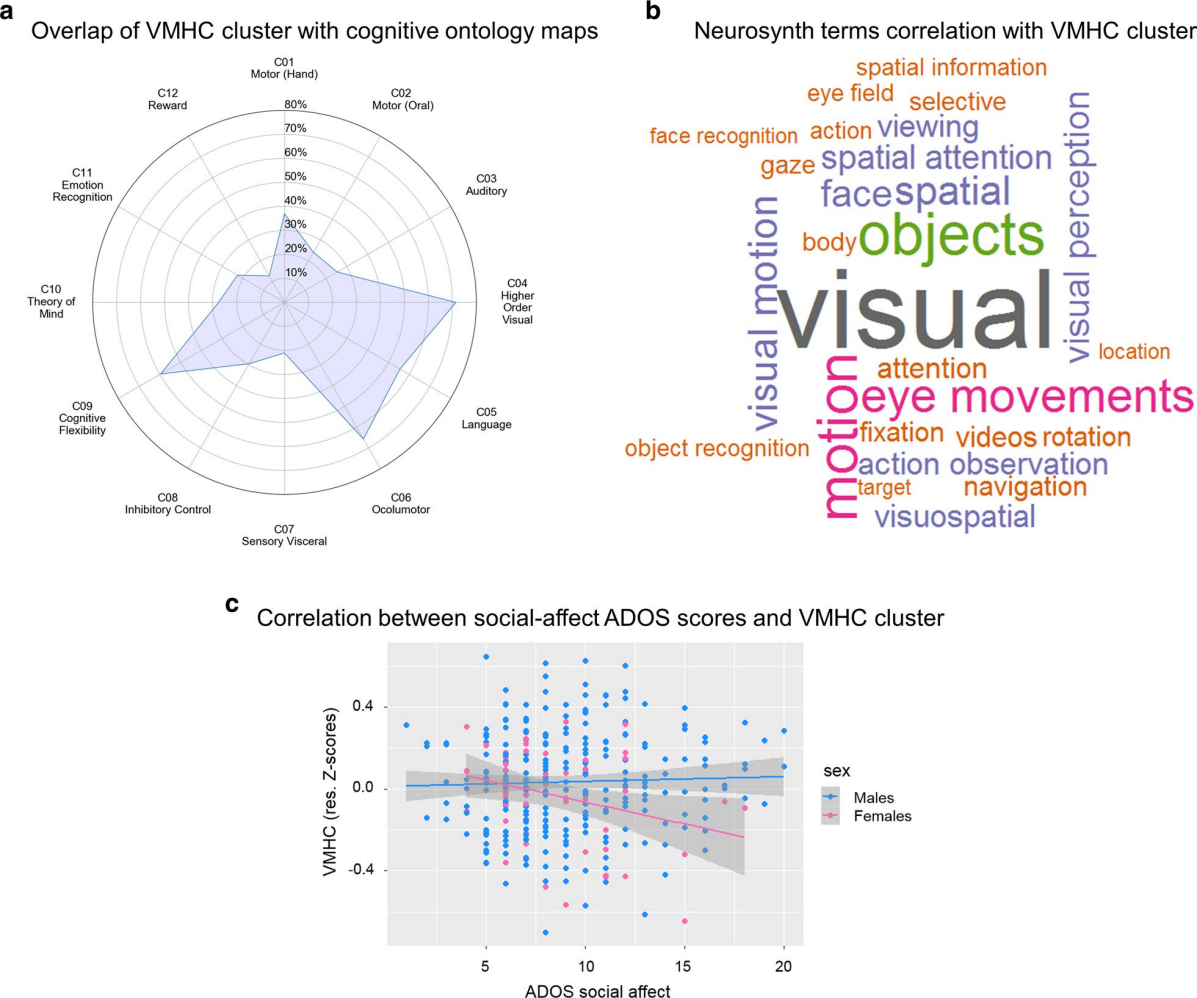
Functional relevance of sex-by-diagnosis interaction in VMHC. **a** The radar plot shows the percentage (0–80%) of overlap between the voxels in the dorsolateral occipital cluster showing a significant VMHC sex-by-diagnosis interaction in discovery analyses and the 12 Yeo cognitive ontology probability maps [63] (probability threshold at *P* = 1e−5) for cognitive components C1–C12. As in Floris et al. [2], we labelled each component based on the top five tasks reported to be most likely recruited by a given component. **b** Word cloud based on the top 27 terms showing correlations between *r* = 0.64 to *r* = 0.10 associated with the same VMHC cluster based on the Neurosynth Image Decoder. **c** Sex-differential association between each individual’s VMHC at the cluster showing a significant sex-by-diagnosis interaction in primary analyses and available ADOS social-affect uncalibrated sub-scores in males and females with ASD. VMHC data are shown as residuals obtained after regressing out mean framewise displacement and age effects. While males showed no significant associations at corrected and uncorrected thresholds, females with lower dorsolateral occipital VMHC showed more severe social-affect symptoms at a uncorrected statistical threshold (*F*_(1,311)_ = 4.44, *p* = 0.036)

Post-hoc analyses to functionally characterize this VMHC sex-by-diagnosis interaction indicated that the VMHC cluster in superior lateral occipital cortex overlapped with cognitive maps involved in higher-order visual, oculomotor, cognitive flexibility and language-related processes (Fig. 3a). Further, as shown in Fig. 3b, the most common terms were primarily related to lower-order visual processing and higher-order visual cognition, such as ‘visuospatial’ and ‘spatial attention.’ To explore potential clinical relevance of the VMHC dorsolateral occipital cluster, we explored brain-behavior relationships as a function of sex, within the autism group using three available ADOS scores (calibrated severity total score, and non-calibrated social affect and RRB subscores; see Additional file 1: Supplementary Methods). Although not surviving a strict Bonferroni correction for multiple testing (i.e., 0.05/3 = 0.02), an interaction effect was observed for ADOS social affect scores. It revealed that more severe social affect deficits (*F*_(1,311)_ = 4.44, *p* = 0.036) were associated with decreased VMHC in females with autism (*r* = − 0.29), but not in males with autism (*r* = 0.03). Given that ABIDE data were aggregated and released when calibrated social affect scores [69] were not available to assess potential differences in language abilities and age, analyses were repeated after including ADOS module (ADOS Module 2 to 4) as a nuisance covariate: results remained unchanged (*F*_(1,306)_ = 5.0, *p* = 0.026) as they did also after removing the few data with the less represented ADOS module 2 (see Additional file 1: Supplementary Methods). There were no significant findings, with regard to the CSS total score and non-calibrated RRB sub-score (Fig. 3c), even at an exploratory statistical threshold of *P* < 0.05.

### Robustness

**Fig. 4:**
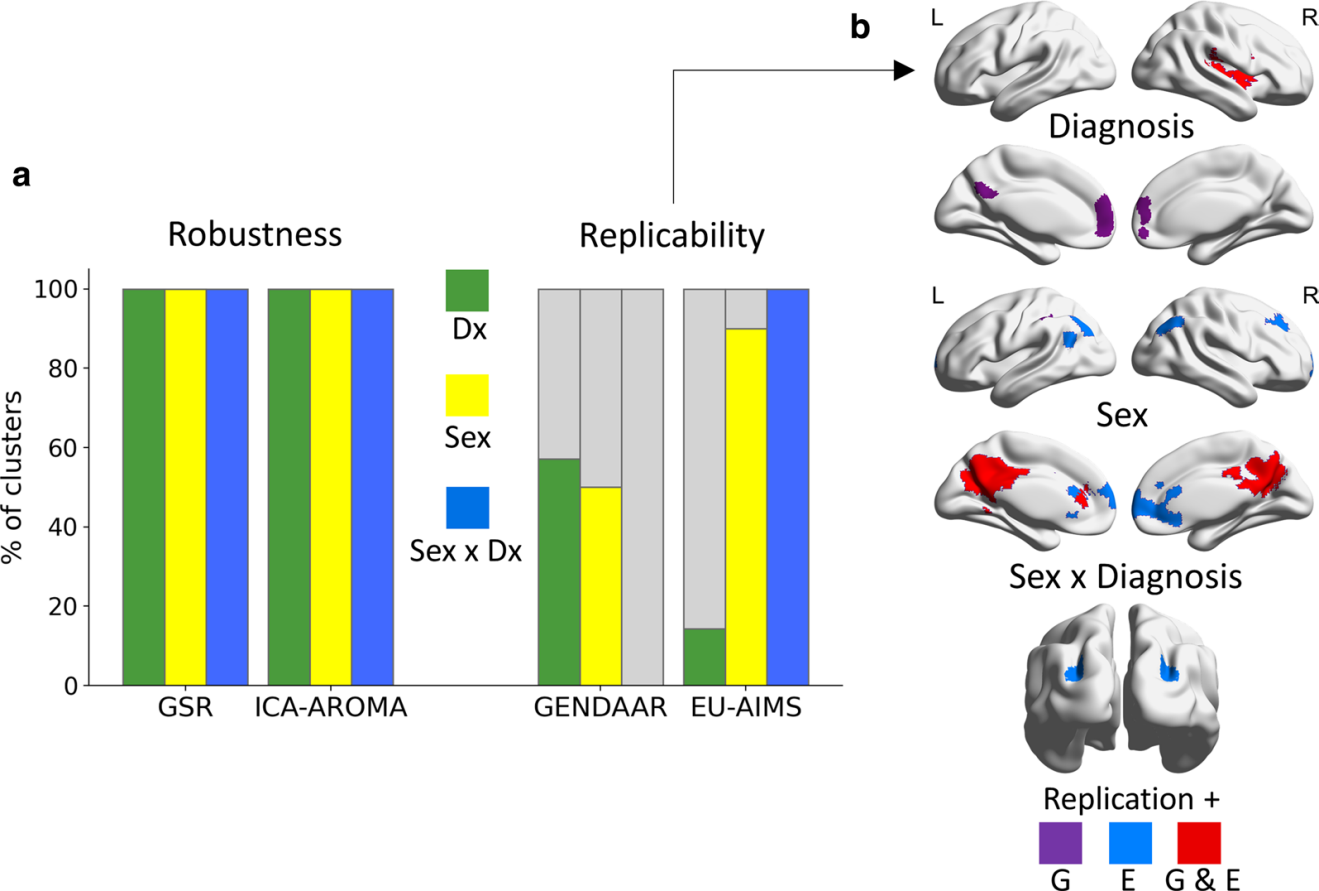
Robustness and replicability summary. **a** The histogram summarizes the percentage of clusters showing a robust and replicable pattern of results as that observed in discovery analyses in the ABIDE sample for main effects of diagnosis (Dx; green; *N* = 7 clusters), sex (yellow; *N* = 10 clusters) and their interaction (blue; *N* = 1 cluster) across three R-fMRI metrics. All findings were robust to different preprocessing pipelines. Across R-fMRI metrics, main sex effects were moderately (50%) to largely (80%) replicable across independent samples: Gender Explorations of Neurogenetics and Development to Advance Autism Research (GENDAAR) and the EU-AIMS Longitudinal European Autism Project (LEAP), respectively. Replicability for main effects of diagnosis was largely replicable in GENDAAR (86%) and minimally replicable in EU-AIMS LEAP (29%). The VMHC pattern observed for sex-by-diagnosis interaction in discovery analyses was replicated in EU-AIMS LEAP only. **b** Surface conjunction maps show the clusters replicated in GENDAAR only (G, purple), EU-AIMS LEAP only (E, blue) and in both samples (G and E, red) for each effect separately

The same pattern of results identified in discovery analyses was observed when the datasets were pre-processed using GSR or ICA-AROMA, across the three R-fMRI metrics in all the clusters identified in the primary analyses across main effects of diagnosis, sex and their interaction; effect size ranges from small to moderate as in discovery analyses (*η*_*p*_^2^ range = 0.01–0.07; Fig. 2b, Fig. 4, Additional file 1: Table S6 and Additional file 3: Figure S6).

### Replicability

#### Main effects of diagnosis

Main effects of diagnosis showed higher replicability (i.e., non-negligible *η*_*p*_^2^ effects showing a similar group mean pattern as observed in discovery analyses) in GENDAAR than in EU-AIMS LEAP. Specifically, across the three R-fMRI metrics that showed significant diagnostic group differences in discovery analyses, six of the seven clusters (86%) in GENDAAR were replicated (*η*_*p*_^2^ range = 0.01–0.04); only two of those seven (29%) were replicated in EU-AIMS LEAP (*η*_*p*_^2^ range = 0.01–0.04). Nevertheless, clusters showing decreased ReHo in ASD versus NT across the insula and central operculum, as well as in the frontal pole were replicated across all samples (Fig. 4, Additional file 1: Table S6 and Additional file 3: Figure S7a).

#### Main effects of sex

Across all three R-fMRI metrics, the main effects of sex observed in the discovery analyses were evident in both independent samples, for most clusters in the EU-AIMS LEAP (80%; 8/10) with effects size ranging from small to moderate (η_p_^2^ range = 0.01–0.06) and for half of the clusters in GENDAAR (50%; 5/10), albeit with small effects (*η*_*p*_^2^ range = 0.01–0.02) (Fig. 4, Additional file 1: Table S6 and Additional file 3: Figure S7b). Notably, the pattern of typical sex differences localized along the default network midline (i.e., decreased VMHC, ReHo and PCC-iFC) was replicated across both independent samples (Fig. 4b).

#### Sex-by-diagnosis interaction effect

The pattern of autism-related VMHC sex differences observed in discovery analyses in the superior lateral occipital cortex was observed in the EU-AIMS LEAP dataset, (*η*_*p*_^2^ = 0.01) (Fig. 2c, Fig. 4, Additional file 1: Table S6). In the GENDAAR dataset, while group means in males with autism, NT males and NT females showed a similar direction as in the ABIDE discovery findings, females with autism differed in magnitude and in the direction of group differences, resulting in a negligible effect with *η*_*p*_^2^ < 0.01 (Fig. 2c).

## Discussion

We examined autism-related sex differences for intrinsic functional brain organization across multiple R-fMRI metrics in a large discovery sample of males and females with autism relative to age-group matched NT selected from the ABIDE repositories [22, 23]. Analyses revealed significant main effects of sex and diagnosis across intrinsic functional connectivity (iFC) of the posterior cingulate cortex, regional homogeneity and voxel-mirrored homotopic connectivity (VMHC) in several cortical regions. Notably, main effects converged along the midline of the default network. In contrast, sex-by-diagnosis interactions were limited to VMHC in the superior lateral occipital cortex. Placed in the context of sex and diagnostic main effects on interhemispheric homotopic connectivity in cortical regions, this result suggests that atypical interhemispheric interactions are pervasive in autism but reflect a combination of sex-independent (i.e., main effect of diagnosis common across sexes) and sex-dependent (i.e., sex-by-diagnosis interaction) effects, each specific to a different functional cortical system. This sex-by-diagnosis interaction effect was robust to distinct pre-processing strategies as those observed for main effects. Further, despite the lack of a priori harmonization for data acquisition among the three samples, this finding was replicable in the larger of the two independent samples (i.e., EU-AIMS LEAP). On one hand, this, together with largely replicable main effects of sex with variable replicability of main diagnostic effects by sample, suggests that inter-sample replicability of R-fMRI can be feasible in autism when sources of variability in diagnostic groups are accounted for in samples sized properly to address such variability. On the other hand, our results highlight the urgent need to obtain multiple harmonized datasets properly powered to systematically address and understand sources of heterogeneity, including and beyond the role of biological sex.

### Sex-dependent and sex-independent atypical interhemispheric interactions in autism

VMHC reflects inter-hemispheric homotopic relations. The strength has been suggested to index coordinated cross-hemispheric processing: *stronger* VMHC indexes weaker hemispheric specialization and vice versa [33, 70]. Several lines of evidence support the notion that the neurobiology of autism is related to atypical hemispheric interactions, including homotopic connectivity and hemispheric lateralization [35, 71–80]. VMHC and functional hemispheric lateralization have also been shown to be sex-differential in NT [33, 81, 82]. The dorsolateral occipital association cortex identified in our discovery analyses is known to serve hemispherically specialized processes, such as visuospatial coordination [83]. Thus, our findings of NT males’ VMHC in dorsolateral occipital cortex being lower than that of NT females are consistent with the notion of increased hemispheric lateralization in this cortical region in NT males relative to NT females. In our data, females with autism instead showed even lower VMHC than NT males, while males with autism showed slightly higher VMHC than NT males. This pattern is indicative of ‘gender-incoherence’ [20] as males and females with autism display the opposite pattern expected in NT per their biological sex. Findings of ‘gender incoherence’ have been reported in earlier neuroimaging studies of autism using different modalities [3, 84, 85]. Among them, several R-fMRI studies explicitly focusing on detecting sex-by-diagnosis interactions (i.e., the regression model included a sex-by-diagnosis interaction term) [3, 11] yielded a pattern of results consistent with ‘gender incoherence.’ In contrast, other studies [12–14] reported a pattern consistent with the ‘extreme male brain’ model [19]—i.e., a shift towards maleness in both females and males with autism. While the seemingly diverging conclusions of these two sets of studies may be attributed to methodological differences, such as the extent of brain networks explored and the statistical modelling employed, findings from our prior work suggest that both shifts towards either maleness or femaleness co-occur in the intrinsic brain of males with autism, in a network-specific manner [2]. However, such prior work did not include female data. Thus, by not directly assessing sex-by-diagnosis interactions, unlike the present study, results could not point to patterns affecting diagnostic differences between the sexes versus those that are common to autism across sexes [4]. This is relevant for efforts focusing on identifying underlying mechanisms. Findings resulting from sex-by-diagnosis interactions may shed light on sex-differential mechanisms that are atypical in autism and may reflect sex-specific susceptibility mechanisms. On the other hand, atypicalities common for both sexes may reflect factors central to the emergence of autism, regardless of whether they overlap with patterns known to be differential between sexes [86]. Interestingly, a recent study based on a sample selected from GENDAAR [16] revealed that the iFC between the nucleus accumbens (selected a priori) and a region of the dorsolateral occipital cortex partially overlapping with that identified by our VMHC analyses, was differentially modulated by the aggregate number of oxytocin receptor risk alleles in females with autism versus NT females and versus males with autism. Although VMHC was not directly tested in the said study [16], its result in dorsolateral occipital cortex is consistent with our observation of atypical sexual differentiation of this visual network region and, together, suggest the need for future whole-brain studies of oxytocin effects in autism.

Along with the sex-dependent autism patterns, our analyses found statistically significant main effects of diagnosis in inter-hemispheric interactions indexed by VMHC in distinct cortical circuits. These were localized along the midline of the DN (paracingulate/frontal cortex consistently and PCC/precuneus) where main effects of PCC-iFC and ReHo also converged. Our results are consistent with prior reports of atypical intrinsic organization of the DN in autism [12, 23, 26, 87–89]. Together they support a common, sex-independent role of DN in autism. This is also highlighted by a recent autism neurosubtyping study that identified three latent iFC factors, all sharing DN atypicalities along with their neurosubtype-specific patterns [90]. Building on this evidence to disentangle the specific role of each of the factors affecting autism in sex-independent and sex-dependent ways, a necessary next step is to engage in novel large-scale data collection efforts including more female data.

### Robustness, replicability and sources of variability

The growing awareness of the replication crisis in neuroscience [91–93] motivated our analyses examining robustness and replicability of findings. While a comprehensive and systematic reproducibility assessment is beyond the scope of the present study, here we focused on examining whether the findings observed in the discovery analyses were also seen after using different preprocessing pipelines—*robustness*—as well as in fully independent, albeit of convenience samples (i.e., not harmonized a priori with each other)—*replicability*. To this end, given the lack of consensus on quantitative metrics of replicability, we opted to use measures of effect size. These are considered complementary to null hypothesis significance testing [94]. In the context of this study, given the use of convenience samples of different sizes, their selection was considered an advantageous and practical means to provide information on the magnitude of group differences in diagnosis and sex, as well as their interaction. Here, we considered findings to be robust and/or replicable for any non-negligible effects (i.e., *η*_*p*_^2^ ≥ 0.01 [95]). We reasoned that given their distributed and heterogenous nature [6], atypicalities in the autism connectome can stem from a combination of differently sized non-negligible effects, as shown for autism in other biological domains such as genetics [96, 97].

With this in mind, across the two preprocessing methods examined here, the patterns of findings were consistent with those observed in discovery analyses across all R-fMRI metrics and effects. These robust results are consistent with a prior study by He and colleagues [46] reporting that differences in a wider range of pre-processing pipelines have marginal effects on variation in diagnostic group average comparisons. Our study confirms and builds on this earlier report by extending findings of robustness to sex group mean differences and their interactions with diagnosis.

A more nuanced picture emerged from the inter-sample analyses as replicability varied by sample, across the effects and R-fMRI metrics examined. Specifically, while inter-sample main effects of sex were moderately to largely replicable across R-fMRI metrics on both independent samples (~ 50 to 80% of the clusters in GENDAAR and EU-AIMS-LEAP, respectively), replicability of diagnostic effects significantly varied by sample (86 to 29%) across R-fMRI metrics. This is at least in part consistent with findings by King et al. [50] who showed that, depending on the R-fMRI feature examined, diagnostic group differences varied across samples. Even in this scenario, King et al. [50] also reported that findings of decreased homotopic connectivity in autism were relatively more stable than other R-fMRI metrics. This observation, combined with the replicability of our VMHC sex-by-diagnosis interaction findings in the larger of the two independent samples (EU-AIMS LEAP), suggests that measures of homotopic connectivity may have specific biological relevance for autism. It is also possible that given the moderate to high test–retest reliability, VMHC is more suitable in efforts assessing replicability [98, 99].

The striking clinical and biological heterogeneity in autism should be considered as a major contributor to discrepancies in findings of studies focusing on the main effects of diagnostic group means contrasts/interactions [100–103]. Against this background, we interpret our replicability findings on diagnostic effects and, in turn, diagnosis-by-sex interactions. Inter-sample differences may have contributed to the more variable results of replicability on the diagnosis main effects. These may include autism symptom level, age, and IQ, albeit secondary analyses suggested that the examined IQ range did not substantially affect the pattern of discovery results. For example, the EU-AIMS LEAP sample was on-average older, had lower VIQ and most notably, lower symptom severity across all subscales of the ADOS and ADI-R than the ABIDE sample. On the other hand, the GENDAAR sample (which has greater number of replicable diagnostic mean group patterns) did not differ from ABIDE in these variables, except for mean age. Furthermore, a fact that is often neglected, is that the NT groups may also present with considerable sample heterogeneity between studies [100, 104]. For instance, our NT controls in the EU-AIMS LEAP sample had lower VIQ than both ABIDE and GENDAAR NT controls. This has potentially influenced the low replicability of diagnosis main effects in EU-AIMS LEAP.

In contrast, sex-by-diagnosis interaction effect on VMHC in the dorsolateral occipital cortex was replicable in the larger sample, the EU-AIMS LEAP, but not in GENDAAR. Small samples introduce larger epistemic variability (i.e., greater variation related to known and unknown confounds) [105]. Increasing the number of subjects/data allows mitigating epistemic variability and, thus, capturing the underlying variability of interest. Thus, although the rate of replicability for the main effect of diagnosis in EU-AIMS LEAP was limited, accounting for biological sex, a known key source variability in autism, may have substantiated a replicable sex-by-diagnosis pattern in this larger sample. In line with sample size concerns, using four datasets sized between 36 and 44 individuals selected from the ABIDE repository, He et al. [46] found low similarity rates of diagnostic group-level differences on the strength of iFC edges in contrast with the largely similar pattern of results across pipelines. Of note, unlike prior efforts [46, 50], we controlled for site effects within each of the samples (i.e., ABIDE, GENDAAR and EU-AIMS LEAP), using ComBat. Future large-scale harmonized data collections are needed to control and assess the impact of inter-sample variability. Taken together, these findings highlight that sample differences can impact replicability.

Beyond clinical and biological sources of variation, samples may differ in MRI acquisition methods, as well as in approaches used to mitigate head motion during data collection and its impact on findings [106]. Adequately controlling for head motion remains a key challenge for future studies assessing inter-sample replicability. For the present study, we excluded individuals with high motion, retained relatively large samples with group average low motion (mean ± standard deviation of mFD range = 0.09–0.16 ± 0.06–0.10 mm), as well as included mFD at the second-level analyses as a nuisance covariate. Overall, the extent to which each sample-related factor affects replicability needs to be systematically examined in future well-powered studies. Only this type of studies will allow for emerging subtyping approaches to dissect heterogeneity by brain imaging features using a range of data-driven methods [107, 108], including normative modelling [72, 109, 110].

Inter-sample differences and methodological differences, beyond nuisance regression, may have contributed to some differences in findings between the present and earlier studies, conducted with independent or partially overlapping samples [11, 23, 41]. For example, Alaerts et al. [11] also examined sex-by-diagnosis interaction in PCC-iFC in a dataset selected from ABIDE I only. Although their pattern of results was consistent with the ‘gender incoherence’ model, the resulting circuit(s) did not involve the dorsolateral occipital cortex as identified with VMHC in the present study. Along with differences in samples selected from the same data repositories, other methodological choices may also affect results*.* For example, prior studies differed with the present one in the inclusion of sex-by-diagnosis interaction [17], the extent of the whole-brain voxel-based analyses [15, 16], or the statistical threshold utilized [23]. Nevertheless, it is remarkable that even in light of these differences, consistent results have emerged including the overarching atypical inter-hemispheric interactions in autism, and sex-dependent and sex-independent atypical intrinsic brain function across distinct functional networks.

## Limitations

Along with the inter-sample differences resulting from the lack of sufficiently available harmonized multi-site replication datasets in the field, other limitations of this study should be addressed in future efforts. One regards the lack of measures differentiating the effects of sex versus those of gender so as to disentangle their relative roles (e.g., gender-identity and gender-expression) in the intrinsic brain properties [111]. Further, in-depth cognitive measures to directly characterize the role of VMHC findings were not available. Additional behavioral measures are needed to establish whether our result in VMHC of the dorsolateral occipital cortex mainly applies to low-level (bottom-up) visual processing differences or higher-level (top-down) attentional/controlled processes in males and females with autism. As a neurodevelopmental condition, autism shows striking inter-individual differences in clinical and developmental trajectories, as well as outcomes. Thus, age may influence symptom presentation [112, 113] and neurobiology [13, 110, 114]. Despite the considerable size of the samples available for this study, it is still difficult to sufficiently cover a broad age range across both males and females and diagnostic groups across contributing sites, and to evaluate age effects appropriately. Even larger cross-sectional samples are needed to derive meaningful age-related information that ultimately requires confirmation in longitudinal studies. Such longitudinal studies would allow to examine the potential impact of puberty and related surge of sex steroid hormones reported in NT boys and girls [115], in autism specifically. Here, we have no direct measure of puberty other than age, but future studies should aim to include such measures. Further, the value of a large-scale and publicly available multi-site resource such as ABIDE also comes with unavoidable site differences which must be considered in data selection, analyses and interpretation of results. Although residual site-related effects may have remained in findings even after using the novel Bayesian approach for correcting for batch-effects, replicability in independent samples suggest that effects are not simply driven by site variability. These results are consistent with earlier reports of reproducible imaging biomarkers even when accounting for inter-site differences in multisite datasets such as the ABIDE I repository [47]. Finally, despite the advantages of effect sizes over p-value when comparing independently collected samples of different sizes and potentially different variances, it is important to acknowledge that they are not without limitations [116] and, thus, should be interpreted with caution. Similar to *p* values, they are influenced by sample sizes and have the equivalent risks of p-hacking. Finally, the standard errors or effect sizes can be large—a concern we addressed by reporting their confidence intervals.

## Conclusions

The present work revealed sex differences in the intrinsic brain of autism, particularly in dorsolateral occipital interhemispheric interactions, which were robust to pre-processing pipeline decisions and replicable in the larger of the two independent samples. While differences in nuisance regression pipelines have little influence on the consistency of findings, sample heterogeneity represents a challenge for replicability of findings. Lateralized cognitive functions and cross-hemispheric interactions should be further explored in relation to sex differences in autism while addressing this challenge with future harmonized data acquisition efforts with even larger samples.

## Supplementary Information

**Additional file 1.** Contains the supplementary method text, supplementary tables S1-S6 and supplementary figures S1-S4.

**Additional file 2.** Stability of main effects of diagnosis and sex.

**Additional file 3.** Robustness and replicability of main effects of diagnosis and sex.

## Data Availability

*ABIDE I and II*: Data are freely accessible in the publicly available Autism Brain Imaging Data Exchange repository (http://fcon_1000.projects.nitrc.org/indi/abide). *EU-AIMS LEAP*: Data are currently only available for sites involved in data collection, but data (starting with the first wave) will be made available on request. *GENDAAR*: Anonymized data are publicly available through the National Database for Autism Research (NDAR) and access can be requested via https://nda.nih.gov/about.html.

## Abbreviations

ABIDE: Autism Brain Imaging Data Exchange
ASD: Autism spectrum disorder
DC: Degree centrality
DN: Default network
EPI: Echo planar image
LEAP: Longitudinal European Autism Project
fALFF: Fractional amplitude of low frequency fluctuations
FIQ: Full-scale IQ
GENDAAR: Gender Explorations of Neurogenetics and Development to Advance Autism Research
GSR: Global Signal Regression
ICA-AROMA: Independent Component Analysis-Automatic Removal of Motion Artifacts
iFC: Intrinsic functional connectivity
mFD: Mean framewise displacement
NDAR: National Database for Autism Research
NT: Neurotypical
PCC: Posterior cingulate cortex
ReHo: Regional homogeneity
R-fMRI: Resting-state functional magnetic resonance imaging
VMHC: Voxel-mirrored homotopic connectivity

## References

[1] Loomes R, Hull L, Mandy WPL (2017). What is the male-to-female ratio in autism spectrum disorder? A systematic review and meta-analysis. J Am Acad Child Adolesc Psychiatry.

[2] Floris DL, Lai MC, Nath T, Milham MP, Di Martino A (2018). Network-specific sex differentiation of intrinsic brain function in males with autism. Mol Autism.

[3] Lai MC, Lombardo MV, Suckling J, Ruigrok ANV, Chakrabarti B, Ecker C (2013). Biological sex affects the neurobiology of autism. Brain.

[4] Lai MC, Lerch JP, Floris DL, Ruigrok ANV, Pohl A, Lombardo MV (2017). Imaging sex/gender and autism in the brain: etiological implications. J Neurosci Res.

[5] Ecker C (2019). Notice of Retraction and Replacement: Ecker et al. Association between the probability of autism spectrum disorder and normative sex-related phenotypic diversity in brain structure. JAMA Psychiatry. 2017;74(4):329-338. JAMA Psychiatry.

[6] Picci G, Gotts SJ, Scherf KS (2016). A theoretical rut: revisiting and critically evaluating the generalized under/over-connectivity hypothesis of autism. Dev Sci.

[7] Geschwind DH, Levitt P (2007). Autism spectrum disorders: developmental disconnection syndromes. Curr Opin Neurobiol.

[8] Smith REW, Avery JA, Wallace GL, Kenworthy L, Gotts SJ, Martin A (2019). Sex differences in resting-state functional connectivity of the cerebellum in autism spectrum disorder. Front Hum Neurosci.

[9] Cummings KK, Lawrence KE, Hernandez LM, Wood ET, Bookheimer SY, Dapretto M (2020). Sex differences in salience network connectivity and its relationship to sensory over-responsivity in youth with autism spectrum disorder. Autism Res.

[10] Henry TR, Dichter GS, Gates K (2018). Age and gender effects on intrinsic connectivity in autism using functional integration and segregation. Biol Psychiatry Cogn Neurosci Neuroimaging.

[11] Alaerts K, Swinnen SP, Wenderoth N (2016). Sex differences in autism: a resting-state fMRI investigation of functional brain connectivity in males and females. Soc Cogn Affect Neurosci.

[12] Ypma RJF, Moseley RL, Holt RJ, Rughooputh N, Floris DL, Chura LR (2016). Default mode hypoconnectivity underlies a sex-related autism spectrum. Biol Psychiatry Cogn Neurosci Neuroimaging.

[13] Kozhemiako N, Vakorin V, Nunes AS, Iarocci G, Ribary U, Doesburg SM (2019). Extreme male developmental trajectories of homotopic brain connectivity in autism. Hum Brain Mapp.

[14] Lee JK, Amaral DG, Solomon M, Rogers SJ, Ozonoff S, Nordahl CW (2020). Sex differences in the amygdala resting-state connectome of children with autism spectrum disorder. Biol Psychiatry Cogn Neurosci Neuroimaging.

[15] Lawrence KE, Hernandez LM, Bowman HC, Padgaonkar NT, Fuster E, Jack A (2020). Sex differences in functional connectivity of the salience, default mode, and central executive networks in youth with ASD. Cereb Cortex.

[16] Hernandez LM, Lawrence KE, Padgaonkar NT, Inada M, Hoekstra JN, Lowe JK (2020). Imaging-genetics of sex differences in ASD: distinct effects of OXTR variants on brain connectivity. Transl Psychiatry.

[17] Kozhemiako N, Nunes AS, Vakorin V, Iarocci G, Ribary U, Doesburg SM (2020). Alterations in local connectivity and their developmental trajectories in autism spectrum disorder: does being female matter?. Cereb Cortex.

[18] Olson LA, Mash LE, Linke A, Fong CH, Müller RA, Fishman I (2020). Sex-related patterns of intrinsic functional connectivity in children and adolescents with autism spectrum disorders. Autism.

[19] Baron-Cohen S (2002). The extreme male brain theory of autism. Trends Cogn Sci.

[20] Bejerot S, Eriksson JM, Bonde S, Carlström K, Humble MB, Eriksson E (2012). The extreme male brain revisited: gender coherence in adults with autism spectrum disorder. Br J Psychiatry.

[21] Watkins EE, Zimmermann ZJ, Poling A (2014). The gender of participants in published research involving people with autism spectrum disorders. Res Autism Spectr Disord.

[22] Di Martino A, O’Connor D, Chen B, Alaerts K, Anderson JS, Assaf M (2017). Enhancing studies of the connectome in autism using the autism brain imaging data exchange II. Sci Data.

[23] Di Martino A, Yan CG, Li Q, Denio E, Castellanos FX, Alaerts K (2014). The autism brain imaging data exchange: towards a large-scale evaluation of the intrinsic brain architecture in autism. Mol Psychiatry.

[24] Yan CG, Yang Z, Colcombe SJ, Zuo XN, Milham MP (2017). Concordance among indices of intrinsic brain function: insights from inter-individual variation and temporal dynamics. Sci Bull.

[25] Di Martino A, Fair DA, Kelly C, Satterthwaite TD, Castellanos FX, Thomason ME (2014). Unraveling the miswired connectome: a developmental perspective. Neuron.

[26] Assaf M, Jagannathan K, Calhoun VD, Miller L, Stevens MC, Sahl R (2010). Abnormal functional connectivity of default mode sub-networks in autism spectrum disorder patients. Neuroimage.

[27] Lynch CJ, Uddin LQ, Supekar K, Khouzam A, Phillips J, Menon V (2013). Default mode network in childhood autism: posteromedial cortex heterogeneity and relationship with social deficits. Biol Psychiatry.

[28] Lau WKW, Leung MK, Lau BWM (2019). Resting-state abnormalities in Autism Spectrum Disorders: a meta-analysis. Sci Rep.

[29] Biswal BB, Mennes M, Zuo XN, Gohel S, Kelly C, Smith SM (2010). Toward discovery science of human brain function. Proc Natl Acad Sci U S A.

[30] Dumais KM, Chernyak S, Nickerson LD, Janes AC (2018). Sex differences in default mode and dorsal attention network engagement. PLoS ONE.

[31] Scheinost D, Finn ES, Tokoglu F, Shen X, Papademetris X, Hampson M (2015). Sex differences in normal age trajectories of functional brain networks. Hum Brain Mapp.

[32] Yan CG, Craddock RC, Zuo XN, Zang YF, Milham MP (2013). Standardizing the intrinsic brain: towards robust measurement of inter-individual variation in 1000 functional connectomes. Neuroimage.

[33] Zuo XN, Kelly C, Di Martino A, Mennes M, Margulies DS, Bangaru S (2010). Growing together and growing apart: regional and sex differences in the lifespan developmental trajectories of functional homotopy. J Neurosci.

[34] Dinstein I, Pierce K, Eyler L, Solso S, Malach R, Behrmann M (2011). Disrupted neural synchronization in toddlers with autism. Neuron.

[35] Hahamy A, Behrmann M, Malach R (2015). The idiosyncratic brain: distortion of spontaneous connectivity patterns in autism spectrum disorder. Nat Neurosci.

[36] Zang Y, Jiang T, Lu Y, He Y, Tian L (2004). Regional homogeneity approach to fMRI data analysis. Neuroimage.

[37] Paakki JJ, Rahko J, Long X, Moilanen I, Tervonen O, Nikkinen J (2010). Alterations in regional homogeneity of resting-state brain activity in autism spectrum disorders. Brain Res.

[38] Shukla DK, Keehn B, Müller RA (2010). Regional homogeneity of fMRI time series in autism spectrum disorders. Neurosci Lett.

[39] Zuo XN, Ehmke R, Mennes M, Imperati D, Castellanos FX, Sporns O (2012). Network centrality in the human functional connectome. Cereb Cortex.

[40] Di Martino A, Zuo XN, Kelly C, Grzadzinski R, Mennes M, Schvarcz A (2013). Shared and distinct intrinsic functional network centrality in autism and attention-deficit/hyperactivity disorder. Biol Psychiatry.

[41] Holiga Š, Hipp JF, Chatham CH, Garces P, Spooren W, D’Ardhuy XL (2019). Patients with autism spectrum disorders display reproducible functional connectivity alterations. Sci Transl Med.

[42] Zou QH, Zhu CZ, Yang Y, Zuo XN, Long XY, Cao QJ (2008). An improved approach to detection of amplitude of low-frequency fluctuation (ALFF) for resting-state fMRI: Fractional ALFF. J Neurosci Methods.

[43] Itahashi T, Yamada T, Watanabe H, Nakamura M, Ohta H, Kanai C (2015). Alterations of local spontaneous brain activity and connectivity in adults with high-functioning autism spectrum disorder. Mol Autism.

[44] Ciric R, Wolf DH, Power JD, Roalf DR, Baum GL, Ruparel K (2017). Benchmarking of participant-level confound regression strategies for the control of motion artifact in studies of functional connectivity. Neuroimage.

[45] Parkes L, Fulcher B, Yücel M, Fornito A (2018). An evaluation of the efficacy, reliability, and sensitivity of motion correction strategies for resting-state functional MRI. Neuroimage.

[46] He Y, Byrge L, Kennedy DP (2020). Nonreplication of functional connectivity differences in autism spectrum disorder across multiple sites and denoising strategies. Hum Brain Mapp.

[47] Abraham A, Milham MP, Di Martino A, Craddock RC, Samaras D, Thirion B (2017). Deriving reproducible biomarkers from multi-site resting-state data: an Autism-based example. Neuroimage.

[48] Alaerts K, Nayar K, Kelly C, Raithel J, Milham MP, Di Martino A (2015). Age-related changes in intrinsic function of the superior temporal sulcus in autism spectrum disorders. Soc Cogn Affect Neurosci.

[49] Yahata N, Morimoto J, Hashimoto R, Lisi G, Shibata K, Kawakubo Y (2016). A small number of abnormal brain connections predicts adult autism spectrum disorder. Nat Commun.

[50] King JB, Prigge MBD, King CK, Morgan J, Weathersby F, Fox JC (2019). Generalizability and reproducibility of functional connectivity in autism. Mol Autism.

[51] Charman T, Loth E, Tillmann J, Crawley D, Wooldridge C, Goyard D (2017). The EU-AIMS Longitudinal European Autism Project (LEAP): clinical characterisation. Mol Autism.

[52] Loth E, Charman T, Mason L, Tillmann J, Jones EJH, Wooldridge C (2017). The EU-AIMS Longitudinal European Autism Project (LEAP): design and methodologies to identify and validate stratification biomarkers for autism spectrum disorders. Mol Autism.

[53] Irimia A, Torgerson CM, Jacokes ZJ, Van Horn JD (2017). The connectomes of males and females with autism spectrum disorder have significantly different white matter connectivity densities. Sci Rep.

[54] Jenkinson M, Bannister P, Brady M, Smith S (2002). Improved optimization for the robust and accurate linear registration and motion correction of brain images. Neuroimage.

[55] Lefebvre A, Beggiato A, Bourgeron T, Toro R (2015). Neuroanatomical diversity of corpus callosum and brain volume in autism: meta-analysis, analysis of the autism brain imaging data exchange project, and simulation. Biol Psychiatry.

[56] Dennis M, Francis DJ, Cirino PT, Schachar R, Barnes MA, Fletcher JMJM (2009). Why IQ is not a covariate in cognitive studies of neurodevelopmental disorders. J Int Neuropsychol Soc.

[57] Friston KJ, Williams S, Howard R, Frackowiak RSJ, Turner R (1996). Movement-related effects in fMRI time-series. Magn Reson Med.

[58] Behzadi Y, Restom K, Liau J, Liu TT (2007). A component based noise correction method (CompCor) for BOLD and perfusion based fMRI. Neuroimage.

[59] Greve DN, Fischl B (2009). Accurate and robust brain image alignment using boundary-based registration. Neuroimage.

[60] Avants BB, Tustison NJ, Wu J, Cook PA, Gee JC (2011). An open source multivariate framework for N-tissue segmentation with evaluation on public data. Neuroinformatics.

[61] Nielson D, Pereira F, Zheng C, Migineishvili N, Lee J, Thomas A, et al. Detecting and harmonizing scanner differences in the ABCD study—annual release 1.0; 2018. Preprint at https://www.biorxiv.org/content/10.1101/309260v1.

[62] Eklund A, Nichols TE, Knutsson H (2016). Cluster failure: why fMRI inferences for spatial extent have inflated false-positive rates. Proc Natl Acad Sci U S A.

[63] Thomas Yeo BT, Krienen FM, Eickhoff SB, Yaakub SN, Fox PT, Buckner RL (2015). Functional specialization and flexibility in human association cortex. Cereb Cortex.

[64] Yarkoni T, Poldrack RA, Nichols TE, Van Essen DC, Wager TD (2011). Large-scale automated synthesis of human functional neuroimaging data. Nat Methods.

[65] Gotham K, Pickles A, Lord C (2009). Standardizing ADOS scores for a measure of severity in autism spectrum disorders. J Autism Dev Disord.

[66] Murphy K, Birn RM, Handwerker DA, Jones TB, Bandettini PA (2009). The impact of global signal regression on resting state correlations: are anti-correlated networks introduced?. Neuroimage.

[67] Pruim RHR, Mennes M, van Rooij D, Llera A, Buitelaar JK, Beckmann CF (2015). ICA-AROMA: a robust ICA-based strategy for removing motion artifacts from fMRI data. Neuroimage.

[68] Kong XZ, Francks C, ENIGMA Laterality Working Group (2020). Reproducibility in the absence of selective reporting: an illustration from large-scale brain asymmetry research. Hum Brain Mapp.

[69] Hus V, Lord C (2014). The autism diagnostic observation schedule, module 4: revised algorithm and standardized severity scores. J Autism Dev Disord.

[70] Stark DE, Margulies DS, Shehzad ZE, Reiss P, Kelly AMC, Uddin LQ (2008). Regional variation in interhemispheric coordination of intrinsic hemodynamic fluctuations. J Neurosci.

[71] Floris DL, Howells H, Forrester GS, Hopkins WD, Hudry K, Lindell AK (2018). Atypical structural and functional motor networks in autism. Progress in brain research: cerebral lateralization and cognition: evolutionary and developmental investigations of behavioral biases.

[72] Floris DL, Wolfers T, Zabihi M, Holz NE, Zwiers MP, Charman T (2020). Atypical brain asymmetry in autism—a candidate for clinically meaningful stratification. Biol Psychiatry Cogn Neurosci Neuroimaging.

[73] Floris DL, Lai MC, Auer T, Lombardo MV, Ecker C, Chakrabarti B (2016). Atypically rightward cerebral asymmetry in male adults with autism stratifies individuals with and without language delay. Hum Brain Mapp.

[74] Floris DL, Chura LR, Holt RJ, Suckling J, Bullmore ET, Baron-Cohen S (2013). Psychological correlates of handedness and corpus callosum asymmetry in autism: the left hemisphere dysfunction theory revisited. J Autism Dev Disord.

[75] Floris DL, Barber AD, Nebel MB, Martinelli M, Lai MC, Crocetti D (2016). Atypical lateralization of motor circuit functional connectivity in children with autism is associated with motor deficits. Mol Autism.

[76] De Fossé L, Hodge SM, Makris N, Kennedy DN, Caviness VS, McGrath L (2004). Language-association cortex asymmetry in autism and specific language impairment. Ann Neurol.

[77] Herbert MR, Ziegler DA, Deutsch CK, O’Brien LM, Kennedy DN, Filipek PA (2005). Brain asymmetries in autism and developmental language disorder: a nested whole-brain analysis. Brain.

[78] Escalante-Mead PR, Minshew NJ, Sweeney JA (2003). Abnormal brain lateralization in high-functioning autism. J Autism Dev Disord.

[79] Flagg EJ, Cardy JEO, Roberts W, Roberts TPL (2005). Language lateralization development in children with autism: insights from the late field magnetoencephalogram. Neurosci Lett.

[80] Lindell AK, Hudry K (2013). Atypicalities in cortical structure, handedness, and functional lateralization for language in autism spectrum disorders. Neuropsychol Rev.

[81] Zaidel E, Aboitiz F, Clarke J (1995). Sexual dimorphism in interhemispheric relations: anatomical-behavioral convergence. Biol Res.

[82] Proverbio AM, Brignone V, Matarazzo S, Del Zotto M, Zani A (2006). Gender differences in hemispheric asymmetry for face processing. BMC Neurosci.

[83] Vogel JJ, Bowers CA, Vogel DS (2003). Cerebral lateralization of spatial abilities: a meta-analysis. Brain Cogn.

[84] Kirkovski M, Enticott PG, Hughes ME, Rossell SL, Fitzgerald PB (2016). Atypical neural activity in males but not females with autism spectrum disorder. J Autism Dev Disord.

[85] Lai MC, Lombardo MV, Chakrabarti B, Ruigrok ANV, Bullmore ET, Suckling J (2019). Neural self-representation in autistic women and association with ‘compensatory camouflaging’. Autism.

[86] Lai MC, Lombardo MV, Auyeung B, Chakrabarti B, Baron-Cohen S (2015). Sex/gender differences and autism: setting the scene for future research. J Am Acad Child Adolesc Psychiatry.

[87] Moseley RL, Ypma RJF, Holt RJ, Floris D, Chura LR, Spencer MD (2015). Whole-brain functional hypoconnectivity as an endophenotype of autism in adolescents. NeuroImage Clin.

[88] Kennedy DP, Courchesne E (2008). Functional abnormalities of the default network during self- and other-reflection in autism. Soc Cogn Affect Neurosci.

[89] Hull JV, Jacokes ZJ, Torgerson CM, Irimia A, Van Horn JD, Aylward E (2017). Resting-state functional connectivity in autism spectrum disorders: a review. Front Psychiatry.

[90] Tang S, Sun N, Floris DL, Zhang X, Di Martino A, Yeo BTTT (2020). Reconciling dimensional and categorical models of autism heterogeneity: a brain connectomics and behavioral study. Biol Psychiatry.

[91] Barch DM, Yarkoni T (2013). Introduction to the special issue on reliability and replication in cognitive and affective neuroscience research. Cogn Affect Behav Neurosci.

[92] Poldrack RA, Poline JB (2015). The publication and reproducibility challenges of shared data. Trends Cogn Sci.

[93] Gorgolewski KJ, Nichols T, Kennedy DN, Poline JB, Poldrack RA (2018). Making replication prestigious. Behav Brain Sci.

[94] Selya AS, Rose JS, Dierker LC, Hedeker D, Mermelstein RJ (2012). A practical guide to calculating Cohen’s f 2, a measure of local effect size, from PROC MIXED. Front Psychol.

[95] Cohen J (1992). Statistical power analysis. Curr Dir Psychol Sci.

[96] Gratten J, Wray NR, Keller MC, Visscher PM (2014). Large-scale genomics unveils the genetic architecture of psychiatric disorders. Nat Neurosci.

[97] Sanders SJ, He X, Willsey AJ, Ercan-Sencicek AG, Samocha KE, Cicek AE (2015). Insights into autism spectrum disorder genomic architecture and biology from 71 risk loci. Neuron.

[98] Zuo XN, Xing XX (2014). Test-retest reliabilities of resting-state FMRI measurements in human brain functional connectomics: a systems neuroscience perspective. Neurosci Biobehav Rev.

[99] Zuo XN, Xu T, Milham MP (2019). Harnessing reliability for neuroscience research. Nat Hum Behav.

[100] Feczko E, Miranda-Dominguez O, Marr M, Graham A, Nigg J, Fair D (2019). The heterogeneity problem: approaches to identify psychiatric subtypes. Trends Cogn Sci.

[101] Marquand AF, Wolfers T, Mennes M, Buitelaar J, Beckmann CF (2016). Beyond lumping and splitting: a review of computational approaches for stratifying psychiatric disorders. Biol Psychiatry Cogn Neurosci Neuroimaging.

[102] Lombardo MV, Lai MC, Baron-Cohen S (2019). Big data approaches to decomposing heterogeneity across the autism spectrum. Mol Psychiatry.

[103] Wolfers T, Floris DL, Dinga R, van Rooij D, Isakoglou C, Kia SM (2019). From pattern classification to stratification: towards conceptualizing the heterogeneity of Autism Spectrum Disorder. Neurosci Biobehav Rev.

[104] Yokota S, Takeuchi H, Hashimoto T, Hashizume H, Asano K, Asano M (2015). Individual differences in cognitive performance and brain structure in typically developing children. Dev Cogn Neurosci.

[105] Marquand AF, Kia SM, Zabihi M, Wolfers T, Buitelaar JK, Beckmann CF (2019). Conceptualizing mental disorders as deviations from normative functioning. Mol Psychiatry.

[106] van Dijk KRA, Sabuncu MR, Buckner RL (2012). The influence of head motion on intrinsic functional connectivity MRI. Neuroimage.

[107] Hong S-J, Vogelstein JT, Gozzi A, Bernhardt BC, Yeo BTT, Milham MP (2020). Towards neurosubtypes in autism. Biol Psychiatry.

[108] Zabihi M, Floris DL, Kia SM, Wolfers T, Tillmann J, Arenas AL (2020). Fractionating autism based on neuroanatomical normative modeling. Transl Psychiatry.

[109] Marquand AF, Rezek I, Buitelaar J, Beckmann CF (2016). Understanding heterogeneity in clinical cohorts using normative models: beyond case-control studies. Biol Psychiatry.

[110] Zabihi M, Oldehinkel M, Wolfers T, Frouin V, Goyard D, Loth E (2019). Dissecting the heterogeneous cortical anatomy of autism spectrum disorder using normative models. Biol Psychiatry Cogn Neurosci Neuroimaging.

[111] Strang JF, van der Miesen AI, Caplan R, Hughes C, DaVanport S, Lai M-C (2020). Both sex- and gender-related factors should be considered in autism research and clinical practice. Autism.

[112] Charman T, Taylor E, Drew A, Cockerill H, Brown JA, Baird G (2005). Outcome at 7 years of children diagnosed with autism at age 2: predictive validity of assessments conducted at 2 and 3 years of age and pattern of symptom change over time. J Child Psychol Psychiatry Allied Discip.

[113] Fecteau S, Mottron L, Berthiaume C, Burack JA (2003). Developmental changes of autistic symptoms. Autism.

[114] Lin HY, Ni HC, Lai MC, Tseng WYI, Gau SSF (2015). Regional brain volume differences between males with and without autism spectrum disorder are highly age-dependent. Mol Autism.

[115] Peper JS, Brouwer RM, Schnack HG, van Baal GC, van Leeuwen M, van den Berg SM (2009). Sex steroids and brain structure in pubertal boys and girls. Psychoneuroendocrinology.

[116] Maher JM, Markey JC, Ebert-May D (2013). The other half of the story: effect size analysis in quantitative research. CBE Life Sci Educ.

