# Supplementary material for: Towards robust and replicable sex differences in the intrinsic brain function of autism
Source: Mol Autism. 2021 Mar 1;12:19. doi: 10.1186/s13229-021-00415-z (PMC7923310; doi:10.1186/s13229-021-00415-z)
Supplement: Supplementary file 1 — Additional file 1. Contains the supplementary method text, supplementary tables S1-S6 and supplementary figures S1-S4. [file 13229_2021_415_MOESM1_ESM.pdf]

Supplementary Material

Towards robust and replicable sex differences in the intrinsic brain of autism

Dorothea L. Floris*, José O. A. Filho*, Meng-Chuan Lai, Steve Giavasis, Marianne Oldehinkel, Maarten Mennes, Tony Charman, Julian Tillmann, Guillaume Dumas, Christine Ecker, Flavio Dell'Acqua, Tobias Banaschewski, Carolin Moessnang, Simon Baron-Cohen, Sarah Durston, Eva Loth, Declan G. M. Murphy, Jan K. Buitelaar, Christian F. Beckmann, Michael P. Milham, Adriana Di Martino

***equal contribution**

This PDF file includes:

1. Supplementary Methods
2. Supplementary Tables (S1 – S6)
3. Supplementary Figures (S1 – S4). Please note that S5 and S6 with S7 are in Additional Files 2 and 3, respectively.

1. Supplementary Methods

1.1. Discovery sample: ABIDE I and II

1.1.1. Inclusion and exclusion criteria

We selected neurotypical (NT) data from individuals without history of any psychiatric disorders (other than specific phobias in neurotypical children), nor of psychotropic medication use. For the autism datasets, autism diagnosis was determined by clinician's consensus supported by either one or both 'gold-standard' diagnostic instruments, i.e., an Autism Diagnostic Observation Schedule, ADOS [1] and/or the Autism Diagnostic Interview-Revised, ADI-R [2] in all sites but two (UCD and Stanford sites only used diagnostic cut-offs of ADOS and/or ADI-R for inclusion). The corresponding MRI data were included in our discovery analyses based on the following selection criteria and visualized in Figure S1: **1)** datasets from sites providing both male and female datasets

(referred to as an individual's imaging data) in at least ten subjects per sex/diagnostic group at age 7-18 years. This age range included the most represented ages across sites, i.e., ages that were present in more than three data collections; **2)** datasets from sites reporting full-scale IQ (FIQ) in at least 75% of individuals per sex/diagnostic group — when missing, FIQ data were estimated by either averaging available performance and verbal IQ scores per sex/diagnostic group or imputing FIQ scores by using the mean per sex/diagnostic group at a given site; **3)** datasets that had more than 95% full-brain coverage; **4)** datasets that successfully completed brain image co-registration and transformation to standard space; **5)** datasets with FIQ scores 2.5 standard deviation (SD) within the mean of the group defined by steps 1 to 4 (mean=109, SD=15.7); **6)** datasets with mean framewise displacement (mFD) [3] within three times the interquartile range (IQR) + the third quartile (Q3) of the group defined by steps 1 to 5 (i.e., mFD <0.39mm; this decision was made due to the non-normal distribution of mFD); **7)** *within* and *across* each site, we assessed diagnostic group age-mean matching by excluding the individual dataset with the highest and lowest age year at each group until group mean matching was reached; **8)** following these steps, males and females within diagnostic groups were matched for FIQ and mFD *within* and *across* sites. We opted not to match between diagnostic groups (i.e., autism and NT), as it would have further limited sample size. Additionally, matching diagnostic groups on FIQ may result in non-representative samples across groups and inclusion of potential confounds (Dennis *et al.*, 2009); **9)** at each step, any sites with less than three individual datasets per diagnostic/sex group were excluded. As a result of this stringent selection process, the final ABIDE I and II sample of N=1,019 included N=82 females with autism, N=362 males with autism, N=166 neurotypical females (NT F), and N=409 neurotypical males (NT M).

1.1.2. Measures of autism severity

Given its specificity [1,4], the Autism Diagnostic Observation Schedule (ADOS) was used to assess autism severity. Total calibrated severity scores (CSS; [5]) were available in the ABIDE I and II data repositories. The CSS range from 1 to 10, with higher scores indicating more severe autism symptom severity. CSS were developed by Gotham et al. [5] to allow comparability across ADOS modules 1–3 which vary by age and language

abilities. All ABIDE I data were collected using the ADOS-G [1] and total CSS were computed post-hoc using the Gotham *et al.*, (2009) guidelines in N=218 (92%) of the autism datasets selected for this study (N=9 sites; KKI_1, NYU, OHSU, PITT, SDSU, STANFORD,UCLA_1, UM_1, YALE). Total CSS were available in all N=207 autism datasets selected for this study from ABIDE II. Among them, N=60 (N=7 sites; GU, KKI_1, NYU, OHSU, SDSU, UCD, UCLA_1) were obtained from scores collected with ADOS-G and converted to CSS based on Gotham's guidelines [5]. The remaining N=149 total CSS (N=8 sites; GU, KKI_1, KKI_2, NYU, OHSU, SDSU, UCD, UCLA_1) were obtained based on ADOS-2 administrations. As a note, across the two ADOS editions, Module 3 was the most used (N=200 in ABIDE I and N=185 in ABIDE II), followed by Module 4 (N=19 in ABIDE I and N=22 in ABIDE II) and Module 2 (N=3 in ABIDE I and N=1 in ABIDE II). Total CSS were used to explore brain behavior relationship. Similar analyses were also conducted with the scaled ADOS subscores for social affect (SA) and restricted repetitive behaviors (RRB) scales. CSS for the subscales SA and RRB scales [4] were not available in ABIDE. These analyses were repeated covarying for ADOS modules 3 and 4 categorically while excluding four individuals where ADOS scores had been collected using Module 2 (due to low number). Results were substantially similar across both analyses

1.2. Replication samples

Both independent samples – the EU-AIMS Longitudinal European Autism Project (EU-AIMS LEAP) and the Gender Explorations of Neurogenetics and Development to Advance Autism Research (GENDAAR) - were based on multisite datasets collected with harmonized MRI and behavioral assessment protocols across sites within each data collection, separately (5 sites for EU-AIMS LEAP and 4 sites for GENDAAR).

As described elsewhere [6], inclusion criteria for the autism group in the **EU-AIMS LEAP** were an existing clinical autism spectrum disorder (ASD) or equivalent diagnosis according to DSM-IV/ICD-10 or DSM-5 criteria. Autism Diagnostic Interview-Revised (ADI-R) [2] and the Autism Diagnostic Observation Schedule, 2nd edition (ADOS-2) also characterizes the sample. Individuals with a clinical ASD diagnosis who did not reach

diagnostic cut-offs on these instruments were not excluded. Individuals with psychosis or bipolar disorder were excluded. For the purpose of this study only datasets from individuals labeled as NT and without history of depression, anxiety, and of psychoactive medication use were included.

For **GENDAAR**, and as described elsewhere [7–9], participants in the autism group were required to have a prior clinical diagnosis of ASD that was confirmed by research-reliable clinicians using the ADOS-2 [10] and/or the ADI-R [2] – notably a protocol was in place to maintain research reliability over time across evaluators between and within sites [8]. Absence of known neurodevelopmental diagnosis and having a first-or second-degree relative with ASD, and no evidence of elevated ASD traits (total t scores < 65 on the parent-report version of the Social Responsiveness Scale, Second Edition [SRS-2]; [11]), were required for NT controls. Further, unaffected siblings of individuals with autism were also excluded. Exclusion criteria for both groups were prematurity, a genetic, neurological or psychiatric comorbidity. These encompass Fragile X syndrome, epilepsy, brain injury, pre-/peri-natal birth injury, nutritional or psychological deprivation, visual or auditory impairment after correction, sensorimotor difficulties, use of any benzodiazepine, barbiturate or anti-epileptic medication, pregnancy and tic disorders.

For both these replication samples, only datasets with more than 95% full-brain coverage after successful anatomical and functional registration were included. Only datasets with age, FIQ in the same range as that of the discovery ABIDE sample (i.e., age=7-18 years; FIQ=70-148) and, similarly, mFD<0.39 were included. For the GENDAAR sample only one member of a sibling pair was included. For a comparison of demographic and clinical information between ABIDE, GENDAAR and EU-AIMS LEAP, see Tables S3, S4 and S5. Briefly, there were significant group mean differences in diagnostic group means for ADOS total CSS (EU-AIMS LEAP < ABIDE = GENDAAR), age (ABIDE < EU-AIMS LEAP = GENDAAR) between the autism groups. In regard to FIQ, significant differences were noted between independent samples, albeit only for the NT groups (ABIDE > EU-AIMS LEAP = GENDAAR).

1.3. R-fMRI measures

Seed-based Correlation Analysis (SCA) was carried out by extracting the mean time series from a spherical region-of-interest mask (8mm in diameter) centered in PCC using the seed location: x=0, y=-53, z=26 previously defined by Andrews-Hanna et al., (2007) [12] and used in Di Martino et al., (2014) [13] and Floris et al., (2018) [14]. Pearson's correlation coefficient was calculated between the PCC time series and each voxel in the brain before being Fisher's z-transformed. PCC-iFC is commonly investigated in autism [13–18] and there are known sex differences in the default network in neurotypicals [8,19–22].

Voxel-Mirrored Homotopic Connectivity (VMHC) [23] is the Pearson's correlation between each voxel and its geometrically corresponding symmetric counterpart in the opposite hemisphere. Spatial transformation parameters were based on a registration to a symmetric MNI template [24] to increase spatial correspondence between homotopic voxels. Correlation coefficients were standardized by applying a Fisher's r-to-z-transformation. Atypical cross-hemispheric homotopic connectivity has previously been reported in autism [13,14,25–28] and shown to differ across the sexes in neurotypicals [23,25].

Regional Homogeneity (ReHo) [29] is a measure of regional coherence between neighboring fMRI time series. It is based on the Kendall's coefficient of concordance [30] between a voxel's time series and its 26 adjacent neighbors. Subject-level maps were transformed into subject-level z-score maps. Previous studies have reported differences in local connectivity in autism [13,14,31–36] and between the sexes in NT [37–39].

Network Degree Centrality (DC) [40] is a measure of local network connectivity. To be consistent with prior studies [13,14,41], here, it is based on a given voxel's sum of significant connections with corresponding $p < 0.001$. DC was calculated based on a study-specific functional volume mask based on voxels (in MNI space) present in at least 90% of subjects and further constrained by a 25% gray matter (GM) probability mask.

Voxel-size was down-sampled to $4mm^3$ to reduce computational intensity. Voxel-based graphs were then generated by computing the Pearson's correlation of each voxel's extracted time series with every other voxel's extracted time series within the study-specific mask. A significance threshold of $p < 0.001$ was applied resulting in a binary, undirected adjacency matrix. DC was then computed by counting the number of significant connections in the adjacency matrix. Subject-level DC-maps were standardized using z-score transformations. Degree centrality is among the graph theoretical measures most commonly used in R-fMRI studies in autism [13,14,42–45] and has been shown to differ across the sexes in NT [40,41].

Fractional Amplitude of Low Frequency Fluctuations (fALFF) [46] is a frequency domain metric representing the relative contribution of specific oscillations to the entire frequency range. It is based on the ratio of the amplitudes of fluctuations in the 0.01-0.1 Hz frequency range to the sum of amplitudes in the entire frequency spectrum. No temporal filtering was applied, because the data were analyzed in the frequency domain. Fractional ALFF maps at the subject-level were transformed into subject-level z-score maps. Previous studies have shown differences in fALFF in individuals with autism compared to neurotypical controls [13,14,45,47,48] and between males and females in NT [37,41,46].

1.4. Preprocessing Pipeline

1.4.1. Structural preprocessing

1) Skull-stripping: T1-weighted images were skull-stripped using FSL's BET [49] command.
2) Tissue segmentation: FSL's FAST [50] command was used to segment images into GM, white matter (WM) and cerebrospinal fluid (CSF). Probability thresholds were 0.96 for WM and CSF, and 0.7 for GM.
3) Spatial normalization: images (with skull-on) were normalized to MNI152 stereotactic space ($2mm^3$ isotropic) with linear and non-linear registrations using

ANTs [51]. For the calculation of VMHC, spatial normalization was done by registering to a symmetrical template.

1.4.2. Functional preprocessing

1) Slice time correction: the AFNI command *3dTshift* was used to correct for differences in acquisition time between the slices using the specific parameters for each site based on their acquisition protocols.
2) Motion realignment: motion correction was performed in two steps. First using the AFNI command *3dvolreg* by each functional volume was co-registered to the (un-aligned) mean functional image. In a second step, a new functional mean image based on the aligned images was used as the reference image. At this second stage, motion parameters based on the Friston 24-Parameter Model (six motion parameters, their values of preceding volumes, 12 squared values of these items) were calculated along with mean framewise displacement (mFD) [3].
3) Skull-stripping: skull was removed using the AFNI command *3dAutomask*.
4) Mean-based intensity normalization: all images were scaled with a factor of 10.000.
5) Nuisance signal regression differed across the discovery and robustness analyses. Across all pipelines 24 motion parameters based on Friston 24-Parameter Model were regressed out along with linear and quadratic trends. For the discovery analysis, the component-based noise correction (CompCor) was used [52]. For robustness analyses two different regression methods were used: Independent Component Analysis - Automatic Removal of Motion Artifacts (ICA-AROMA) [53] and global signal regression (GSR). Details of each of these methods are addressed below.
6) Temporal filtering: band-pass filtering (0.01-0.1 Hz) was done using the AFNI command *3dBandpass* for all R-fMRI derivatives other than fALFF.
7) Registration: functional-to-anatomical co-registration was achieved by Boundary Based Registration (BBR) [54] using FSL FLIRT. Spatial normalization of

functional EPIs to MNI152 space was done by applying linear and non-linear transforms from ANTs.

8) ReHo, fALFF, and SCA of PCC were calculated in native space, before being transformed into MNI152 space. DC was calculated in MNI152 space. As above, VMHC was calculated based on smoothed data in symmetric MNI152 space.
9) Spatial filtering: Derivatives (fALFF, ReHo, SCA, DC) were smoothed with a 3D Gaussian kernel (FWHM=6mm) after computing and registering each derivative. VMHC was spatially filtered (FWHM=6mm) prior to its calculation and registration.

1.5. ComBat

Given that the discovery and replication samples were all multisite, to account for site and collection time variability we applied ComBat (https://github.com/brentp/combat.py) [23]. ComBat harmonization is a statistical technique originally designed for genomic studies to correct unwanted non-biological variability across multiple batches of gene expression microarray experiments [55], also called "batch effects." In genomic studies, these batch effects are systematically introduced when new samples are added to an existing dataset of arrays or in multiple studies that make use of microarrays data across different labs. In the context of MRI images, the batch effects can be introduced by differences in the scanner manufacturers, differences in data collection timing and sites. Recent studies demonstrated that ComBat is a robust method to correct confounding differences on multi-site DTI [56], cortical thickness [57] and fMRI [58] datasets. ComBat uses a parametrical and non-parametrical empirical Bayes framework defined by the following location and scale (L/S) batch adjustment model:

$$Y_{ijv} = a_v + X\beta_v + \gamma_{iv} + \delta_{iv}\varepsilon_{ijv}$$

where Y_{ijv} represents the expression value for voxel v for sample j from site i, a_v is the Z-score value for voxel v, X represents the design matrix for nuisance signals, and β_v is the vector of regression coefficients corresponding to X. The error term ε_{ijv} is assumed to have a normal distribution with zero mean and variance σ_v^2. The parameters γ_{iv} and δ_{iv} are the additive and multiplicative site effects i for voxel v, respectively, and both are

estimated empirically by assuming that all voxels share the same common distribution [55,56].

1.6. Robustness analyses

In discovery analyses on the ABIDE datasets, nuisance signal regression was performed using component-based noise correction (CompCor) [52] to remove physiological noise. Using CompCor the first 5 principal components from a combined WM/CSF mask are regressed out at each individual general linear model. To assess the robustness of the results obtained in discovery analyses to changes in preprocessing approaches, we repeated the analyses replacing the CompCor step – with either ICA-AROMA [53] or GSR [59,60] as nuisance regressions approaches. All other structural and functional preprocessing steps remained unchanged. For both the ICA-AROMA and GSR regression pipelines, WM and CSF signals were also regressed.

ICA-AROMA. It aims to reduce motion-induced signals variation in R-fMRI. This technique largely preserves the autocorrelation structure of the fMRI time-series and it also avoids the reduction of the temporal degrees of freedom, which are known drawback of scrubbing and regressing of motion volumes techniques [53]. ICA-AROMA steps include: 1) automatic estimation and extraction of independent components (ICs) implemented in the FSL's probabilistic ICA tool, MELODIC [61], 2) automatic motion-related ICs classification based on a combination of four discriminative features that represent motion artifacts: high-frequency content features, realignment parameters and spatial features consisting of edge and CSF fraction metrics, and 3) removal of the classified ICs from the fMRI data using linear regression (Pruim et al., 2015).

GSR. Global signal regression (GSR) involves the averaging of all voxel time-series within a gray matter / whole brain mask and its inclusion in the general linear model analysis as a nuisance regressor. For this study, GSR was computed based on the gray matter mask available in CPAC. As a time-varying spatial average, GSR is thought to improve the detection of neuronal signals by removing other sources of global variance (e.g., motion and respiratory-related artifacts) [62,63]. However, GSR has also been

reported to alter functional connectivity maps by shifting the distribution of iFC values and introducing negative correlations [64]. As the discussion on whether to use the global signal as a nuisance regressor has been ongoing in the fMRI community, we opted to also include it as an approach for the robustness strategy.

All preprocessing pipelines were conducted using the Configurable Pipeline for the Analysis of Connectomes (C-PAC, http://fcp-indi.github.com/C-PAC/). CPAC version 0.3.9 was used for all analyses with the exception of those focusing on ICA-AROMA, which was not implemented until V1.30. Regression-testing performed at the time of each CPAC version release was used to confirm that there were no appreciable changes between CPAC versions in any key intermediates (e.g., nuisance covariates) or derived data calculated (i.e., concordance correlation coefficient > 0.98 against an established reference output benchmark).

2. Supplementary Tables

Table S1. Characterization of EU-AIMS LEAP sample

EU-AIMS LEAP	Sites[a]	ASD M (N=133)	ASD F (N=43)	NT M (N=85)	NT F (N=48)		
	**N**	**Mean (SD) [Range]**	**Mean (SD) [Range]**	**Mean (SD) [Range]**	**Mean (SD) [Range]**	**Statistics**	**Post-hoc**
**Age**	5	14.2 (3.0) [7.5- 18.9]	13.3 (3.4) [7.1-18.9]	13.9 (3.0) [7.6- 18.8]	13.9 (3.8) [6.9- 18.6]	$F_{(3)}$=0.83 p=0.48	
**Full-Scale IQ[b]**	5	103 (16) [72-148]	101 (15.9) [70.4-131]	108 (15) [71.7-140]	107 (13.2) [72.7-133]	$F_{(3)}$=2.59 p=0.05	(ASD M=ASD F) < (NT M=NT F)
**Verbal IQ[b]**	5	102 (16.9) [67-144]	100 (15) [67-136]	106 (15.4) [73-142]	105 (14.9) [65-140]	$F_{(3)}$=2.11 p=0.1	
**Performance IQ[b]**	5	105 (17.9) [59-150]	101 (18.7) [58-133]	109 (17.7) [61-139]	107 (14.4) [70-139]	$F_{(3)}$=1.85 p=0.14	
**Mean FD**	5	0.13 (0.08) [0.02-0.35]	0.1 (0.07) [0.04-0.33]	0.1 (0.07) [0.03-0.31]	0.09 (0.07) [0.02-0.36]	$H_{(3)}$=15.88 p=0.001	(ASD M>ASD F) < (NT M=NT F)
**ADI-R**							
**Social**	5	17.2 (6.3) [1-29]	16.1 (7.8) [1-27]	-	-	$t_{(62)}$=0.84 p=0.41	
**Communication**	5	13.7 (5.5) [2-26]	12.8 (5.6) [1-24]	-	-	$t_{(71)}$=0.93 p=0.36	
**RRB**	5	4.5 (2.8) [0-12]	4.4 (2.7) [0-10]	-	-	$t_{(74)}$=0.23 p=0.82	
**ADOS-2**							
**Social-Affect CSS[c]**	4	6.2 (2.6) [1-10]	5.4 (2.6) [1-10]	-	-	$t_{(70)}$=1.65 p=0.1	
**RRB CSS[c]**	4	4.6 (2.7) [1-10]	4.6 (2.5) [1-9]	-	-	$t_{(77)}$=-0.01 p=0.99	
**CSS total[d]**	4	5.5 (2.8) [1-10]	4.5 (2.6) [1-9]	-	-	$t_{(76)}$=2.08 p=0.04	
		**N**	**N**			**Statistics**	**Post-hoc**

**Comorbidity**	4	52[e]	29[f]	-	-	$\chi^2_{(1)}$=9.4 *p*=0.002
**Psychoactive Medication**	4	102	25	-	-	$\chi^2_{(1)}$=4.7 *p*=0.03

Abbreviations: *ADI-R* = Autism Diagnostic Interview-Revised; *ADOS-2* = Autism Diagnostic Observation Schedule-2; *ASD* = Autism Spectrum Disorder; *CSS* = Calibrated Severity Score*; F* = females; *IQ* = intellectual quotient; *M* = males; *Mean FD* = mean framewise displacement [3]; *NT* = neurotypical; RRB= restricted repetitive behaviors. [a]EU-AIMS LEAP data collections: Kings College London, UK, Cambridge University, UK; Donders Institute Nijmegen, Netherlands; University of Utrecht, Netherlands; ZI Mannheim, Germany; [b]FIQ, VIQ and PIQ were assessed using the WASI or WISC / WAIS; [c] Social-Affect & RRB Calibrated Severity Scores computed based on [4] for Module 3 and [65] for Module 4; [d]Total Calibrated Severity Score computed based on [5] for Module 3 and based on [65] for Module 4; [e]ADHD (N=49); anxiety disorder (N=5); depression (N=5). [f]ADHD (N=21); anxiety disorder (N=3); depression (N=2). The three group means were compared with ANOVA tests (or Kruskal-Wallis test in the case of non-parametric mean FD) followed by post-hoc pairwise t-test comparisons (or Mann-Whitney U-tests in the case of non-parametric mean FD) when statistically significant (significance cut-off set at p<0.05).

Table S2. Characterization of GENDAAR sample

GENDAAR	Sites[a]	ASD M (N=43)	ASD F (N=44)	NT M (N=56)	NT F (N=53)		
	N	Mean (SD) [Range]	Mean (SD) [Range]	Mean (SD) [Range]	Mean (SD) [Range]	Statistics	Post-hoc
**Age**	4	13.4 (3.0) [8.2- 17.9]	13.6 (2.7) [8.2-18.0]	13.7 (2.7) [8.4- 17.8]	13.7 (2.8) [8.2- 17.9]	$F_{(3)}$=1.22 p=0.93	
**Full-Scale IQ[b]**	4	101 (17.2) [71-139]	102 (19.9) [70-145]	112 (14.8) [79-143]	111 (14.0) [83-139]	$F_{(3)}$=5.86 p<0.001	(ASD M=ASD F) < (NT M=NT F)
**Verbal IQ[c]**	4	102 (20.5) [52-152]	103 (19.2) [63-147]	112 (17.3) [74-159]	110 (14.0) [84-138]	$F_{(3)}$=3.8 p=0.011	
**Performance IQ[d]**	4	101 (15.2) [61-136]	101 (19.6) [66-143]	111 (13.2) [85-136]	109 (13.7) [84-139]	$F_{(3)}$=2.57 p=0.06	
**Mean FD**	4	0.16 (0.1) [0.03-0.39]	0.16 (0.11) [0.03-0.37]	0.13 (0.09) [0.03-0.36]	0.11 (0.1) [0.02-0.39]	$H_{(3)}$=11.6 p=0.008	(ASD M=ASD F) < (NT M=NT F)
**ADI-R**							
**Social**	4	19.6 (5.6) [5-27]	19.0 (6.0) [1-30]	-	-	$t_{(82)}$=0.49, p=0.63	
**Communication**	4	17.0 (4.2) [8-26]	15.5 (4.6) 4-24]	-	-	$t_{(81)}$=1.56 p=0.12	
**RRB**	4	6.0 (2.6) [1-12]	5.9 (3.1) [0-12]	-	-	$t_{(80)}$=0.15 p=0.88	
**ADOS-2**							
**Social-Affect**	4	10.1 (4.8) [0-19]	7.9 (3.5) [1-18]	-	-	$t_{(59)}$=2.19 p=0.03	ASD M>ASD F
**RRB**	4	2.9 (1.7) [0-6]	2.0 (1.8) [0-6]	-	-	$t_{(71)}$=1.97 p=0.05	ASD M>ASD F
**CSS total**	4	7.3 (2.4) [1-10]	6 (2.3) [1-10]	-	-	$t_{(64)}$=2.37 p=0.02	ASD M>ASD F

ADI-R = Autism Diagnostic Interview-Revised; *ADOS-2* = Autism Diagnostic Observation Schedule-2; *ASD* = Autism Spectrum Disorder; *CSS* = Calibrated Severity Score*;* *F* = females; *GENDAAR*= Gender Explorations of Neurogenetics and Development to Advance Autism Research; *IQ* = intellectual quotient; *M* = males; *Mean FD* = mean framewise displacement [3]; *NT* = neurotypical; RRB= restricted repetitive behaviors. [a]GENDAAR data collections: The Nelson Laboratory of

Cognitive Neuroscience, Boston Children's Hospital, Harvard Medical School, Boston, MA; the Center on Human Development & Disability, Seattle Children's Hospital, University of Washington School of Medicine, Seattle, WA; Staglin IMHRO Center for Cognitive Neuroscience, David Geffen School of Medicine, University of California, Los Angeles, CA; [b]FIQ, VIQ and PIQ (Non-Verbal Reasoning) were assessed using the Differential Ability Scales (DAS II). The three group means were compared with ANOVA tests (or Kruskal-Wallis test in the case of non-parametric mean FD) followed by post-hoc pairwise t-test comparisons (or Mann-Whitney U-tests in the case of non-parametric mean FD) when statistically significant (significance cut-off set at $p<0.05$).

Table S3. Comparison between ABIDE vs. EU-AIMS LEAP sample

	ASD M$_{ABIDE}$ vs. ASD M$_{EU-AIMS LEAP}$		ASD F$_{ABIDE}$ vs. ASD F$_{EU-AIMS LEAP}$		NT M$_{ABIDE}$ vs. NT M$_{EU-AIMS LEAP}$		NT F$_{ABIDE}$ vs. NT F$_{EU-AIMS LEAP}$	
**Age**	$t_{(208)}$=-7.9 p<0.001	**A < E**	$t_{(71)}$=-2.6 p=0.01	**A < E**	$t_{(112)}$=-6.2 p<0.001	**A < E**	$t_{(57)}$=-4.5 p<0.001	**A < E**
**FIQ**	$t_{(238)}$=1.8 p=0.08	A = E	$t_{(87)}$=0.9 p=0.34	A = E	$t_{(109)}$=2.5 p=0.01*	**A > E**	$t_{(74)}$=3.3 p=0.001*	**A > E**
**VIQ**	$t_{(254)}$=2.9 p<0.01*	**A > E**	$t_{(99)}$=1.6 p=0.12	A = E	$t_{(115)}$=4.4 p<0.001*	**A > E**	$t_{(80)}$=3.5 p<0.001*	**A > E**
**PIQ**	$t_{(237)}$=0.6 p=0.55	A = E	$t_{(84)}$=0.8 p=0.44	A = E	$t_{(110)}$=-0.11 p=0.91	A = E	$t_{(76)}$=1.1 p=0.29	A = E
**Mean FD**	U=21380 p=0.06	A = E	U=2189 p=0.03*	**A > E**	U=16233 p=0.34	A = E	U=4136 p=0.69	A = E
**ADOS CSS total**	$t_{(183)}$=4.8 p<0.001*	**A > E**	$t_{(65)}$=5.3 p<0.001*	**A > E**	-	-	-	-
**ADI-R social**	$t_{(201)}$=3.9 p<0.001*	**A > E**	$t_{(70)}$=2.5 p=0.01*	**A > E**	-	-	-	-
**ADI-R comm**	$t_{(202)}$=3.4 p<0.001*	**A > E**	$t_{(83)}$=2.2 p<0.03*	**A > E**	-	-	-	-
**ADI-R RRB**	$t_{(209)}$=5.2 p<0.001*	**A > E**	$t_{(84)}$=2.7 p=0.01*	**A > E**	-	-	-	-

Abbreviations: A = Autism Brain Imaging Data Exchange (ABIDE)*; ADI-R* = Autism Diagnostic Interview-Revised; *ADI-R comm* = ADI-R total communication subscore; ADI-R RRB = ADI-R total restricted repetitive behaviors subscore; *ADOS* = Autism Diagnostic Observation Schedule; *ASD* = Autism Spectrum Disorder; *CSS* = Calibrated Severity Score*; E*= EU-AIMS Longitudinal European Autism Project (EU-AIMS LEAP); *F* = females; *IQ* = intellectual quotient; *FIQ*= full IQ; *VIQ* = verbal IQ; *PIQ* = performance IQ; *M* = males; *Mean FD* = mean framewise displacement [3]; *NT* = neurotypical. See Table 1 and Supplemental Table 1 for group means and SD and Supplemental Material for inclusion, exclusion and study selection criteria for these datasets. * indicate statistically significant with two-tailed tests setting a significance cut-off at p<0.05. Due to non-normal distribution of Mean FD, a non-parametric Man-Whitney U test was used in this case.

Table S4. Comparison between ABIDE vs. GENDAAR sample

	ASD M_{ABIDE} vs. ASD $M_{GENDAAR}$		ASD F_{ABIDE} vs. ASD $F_{GENDAAR}$		NT M_{ABIDE} vs. NT $M_{GENDAAR}$		NT F_{ABIDE} vs. NT $F_{GENDAAR}$	
**Age**	$t_{(50)}$=-3.3 p<0.01*	**A < G**	$t_{(88)}$=-3.7 p<0.001*	**A < G**	$t_{(70)}$=-5.1 p<0.001*	**A < G**	$t_{(74)}$=-5.4 p<0.001*	**A < G**
**FIQ**	$t_{(52)}$=1.8 p<0.07	A = G	$t_{(75)}$=0.6 p=0.55	A = G	$t_{(67)}$=0.2 p=0.87	A = G	$t_{(77)}$=1.3 p=0.2	A = G
**VIQ**	$t_{(51)}$=1.4 p=0.15	A = G	$t_{(84)}$=0.6 p=0.54	A = G	$t_{(66)}$=1.0 p=0.34	A = G	$t_{(92)}$=1.7 p=0.1	A = G
**PIQ**	$t_{(53)}$=1.2 p=0.23	A = G	$t_{(85)}$=0.6 p=0.54	A = G	$t_{(74)}$=-0.5 p=0.64	A = G	$t_{(77)}$=0.4 p=0.65	A = G
**Mean FD**	U=5274 p<0.001*	**A < G**	U=1649 p=0.42	A = G	U=9028 p=0.01*	**A < G**	U=4067 p=0.41	A = G
**ADOS CSS total**	$t_{(35)}$=-1.1 p=0.23	A = G	$t_{(55)}$=1.9 p=0.06	A = G	-	-	-	-
**ADI-R social**	$t_{(51)}$=0.06 p=0.94	A = G	$t_{(83)}$=0.5 p=0.62	A = G	-	-	-	-
**ADI-R comm**	$t_{(54)}$=-2.03 p=0.05	A = G	$t_{(93)}$=-0.35 p=0.73	A = G	-	-	-	-
**ADI-R RRB**	$t_{(51)}$=0.01 p=0.99	A = G	$t_{(74)}$=-0.13 p=0.9	A = G	-	-	-	-

Abbreviations: A = Autism brain imaging data exchange (ABIDE)*; ADI-R* = Autism Diagnostic Interview-Revised; *ADI-R comm* = ADI-R total communication subscore; ADI-R RRB = ADI-R total restricted repetitive behaviors subscore; *ADOS* = Autism Diagnostic Observation Schedule; *ASD* = Autism Spectrum Disorder; *CSS* = Calibrated Severity Score*; F* = females; *IQ* = intellectual quotient; *FIQ*= full IQ; *G*= Gender Explorations of Neurogenetics and Development to Advance Autism Research (GENDAAR); *VIQ* = verbal IQ; *PIQ* = performance IQ; *M* = males; *Mean FD* = mean framewise displacement [3]; *NT* = neurotypical. See Table 1 and Supplemental Table 2 for group means and SD and Supplementary Material for inclusion, exclusion and study selection criteria for these datasets. * indicate statistically significant with two-tailed tests setting a significance cut-off at p<0.05. Due to non-normal distribution of Mean FD, a non-parametric Man-Whitney U test was used in this case.

Table S5. Comparison between EU-AIMS LEAP vs. GENDAAR sample

	ASD M EU-AIMS LEAP vs. ASD M GENDAAR		ASD F EU-AIMS LEAP vs. ASD F GENDAAR		NT M EU-AIMS LEAP vs. NT M GENDAAR		NT F EU-AIMS LEAP vs. NT F GENDAAR	
**Age**	$t_{(72)}$=1.5 p=0.14	E = G	$t_{(80)}$=-0.5 p=0.62	E = G	$t_{(127)}$=0.4 p=0.67	E = G	$t_{(87)}$=0.3 p=0.11	E = G
**FIQ**	$t_{(68)}$=0.7 p=0.48	E = G	$t_{(82)}$=-0.2 p=0.83	E = G	$t_{(120)}$=-1.6 p=0.11	E = G	$t_{(96)}$=-1.7 p=0.1	E = G
**VIQ**	$t_{(62)}$=-0.1 p=0.88	E = G	$t_{(81)}$=-0.7 p=0.48	E = G	$t_{(109)}$=-2.0 p=0.04*	**E < G**	$t_{(96)}$=1.7 p=0.1	E = G
**PIQ**	$t_{(71)}$=0.8 p=0.44	E = G	$t_{(85)}$=-0.1 p=0.89	E = G	$t_{(133)}$=-0.3 p=0.79	E = G	$t_{(97)}$=-0.5 p=0.64	E = G
**Mean FD**	U=2265 p=0.04*	**E < G**	U=660 p=0.02*	**E < G**	U=2065 p=0.18	E = G	U=1126 p=0.32	E = G
**ADOS CSS total**	$t_{(53)}$=-3.7 p<0.001*	**E < G**	$t_{(76)}$=-2.8 p<0.01*	**E < G**	-	-	-	-
**ADI-R social**	$t_{(78)}$=-2.3 p=0.02*	**E < G**	$t_{(79)}$=-2.0 p=0.05*	**E < G**	-	-	-	-
**ADI-R comm**	$t_{(90)}$=-4.0 p<0.001*	**E < G**	$t_{(81)}$=-2.5 p=0.01*	**E < G**	-	-	-	-
**ADI-R RRB**	$t_{(74)}$=3.1 p<0.01*	**E < G**	$t_{(83)}$=2.5 p=0.02*	**E < G**	-	-	-	-

Abbreviations: ADI-R = Autism Diagnostic Interview-Revised; *ADI-R comm* = ADI-R total communication subscore; ADI-R RRB = ADI-R total restricted repetitive behaviors subscore; *ADOS* = Autism Diagnostic Observation Schedule; *ASD* = Autism Spectrum Disorder; *CSS* = Calibrated Severity Score*; E* = EU-AIMS Longitudinal European Autism Project (EU-AIMS LEAP); *F* = females; *IQ* = intellectual quotient; *FIQ*= full IQ; *G*= Gender Explorations of Neurogenetics and Development to Advance Autism Research (GENDAAR); *VIQ* = verbal IQ; *PIQ* = performance IQ; *M* = males; *Mean FD* = mean framewise displacement [3]; *NT* = neurotypical. See Supplemental Tables 1 and 2 for group means and SD and supplementary Material for inclusion, exclusion and study selection criteria for these datasets. * indicate statistically significant with two-tailed tests setting a significance cut-off at p<0.05. Due to non-normal distribution of Mean FD, a non-parametric Man-Whitney U test was used in this case.

Table S6 - Clusters with significant main effect of diagnosis and sex for robustness, replicability, and sex by diagnosis interaction

							DISCOVERY			ROBUSTNESS						REPLICABILITY					
R-fMRI metric	Cluster		Center of Gravity (MNI)			Statistic	CompCor (N=1019)			GSR (N=1019)			ICA AROMA (N=1019)			GENDAAR (N=196)			EU-AIMS (N=309)		
	Anatomical Label	# of voxels	x	y	z	Z	η_p^2	CI-	CI+	η_p^2	CI-	CI+	η_p^2	CI-	CI+	η_p^2	CI-	CI+	η_p^2	CI-	CI+
**DIAGNOSIS**																					
PCC-iFC	PCG/FP	743	-2	56	6	3.9	0.03	0.01	0.05	0.03	0.00	0.01	0.01	0.00	0.03	0.01	0.00	0.06	0.00	0.00	0.00
	sLOC	567	36	76	-76	3.8	0.02	0.01	0.05	0.03	0.00	0.03	0.01	0.00	0.02	0.00	0.00	0.01	0.00	0.00	0.00
	TOFC/OFG	529	-40	-60	-16	4.4	0.01	0.00	0.03	0.01	0.00	0.01	0.01	0.00	0.03	0.01	0.00	0.05	0.00	0.00	0.01
VMHC*	PCC/Prec	191	-6	-54	30	4.2	0.03	0.01	0.05	0.04	0.02	0.07	0.01	0.00	0.03	0.03	0.00	0.09	0.00	0.00	0.00
	PCG/FP	276	-4	54	14	3.9	0.03	0.01	0.05	0.02	0.01	0.04	0.01	0.00	0.03	0.01	0.00	0.05	0.00	0.00	0.00
ReHo	PCG/FP	1072	-2	54	10	5.2	0.01	0.00	0.03	0.02	0.01	0.05	0.02	0.01	0.04	0.04	0.00	0.11	0.01	0.00	0.05
	CO/Ins	1131	44	-8	8	3.4	0.01	0.00	0.03	0.03	0.01	0.06	0.02	0.00	0.03	0.01	0.00	0.05	0.04	0.01	0.09
**SEX**																					
PCC-iFC	MFG	676	28	32	42	3.4	0.03	0.01	0.06	0.03	0.01	0.05	0.02	0.01	0.04	0.00	0.00	0.03	0.01	0.00	0.04
	sLOC	901	46	-66	42	5.2	0.04	0.02	0.07	0.03	0.02	0.06	0.04	0.00	0.03	0.00	0.00	0.04	0.03	0.00	0.08
	sLOC	966	-40	-74	40	6.7	0.05	0.03	0.08	0.04	0.02	0.07	0.03	0.01	0.05	0.00	0.00	0.04	0.03	0.00	0.07
	PCG/FP	2261	4	50	8	3.3	0.04	0.02	0.07	0.04	0.02	0.07	0.02	0.01	0.04	0.01	0.00	0.05	0.03	0.00	0.07
	PCC/Prec	4385	2	-54	32	4.4	0.04	0.02	0.07	0.04	0.02	0.07	0.02	0.01	0.04	0.01	0.00	0.05	0.03	0.00	0.08
VMHC*	ACC	355	-2	24	24	3.6	0.05	0.02	0.07	0.03	0.01	0.05	0.02	0.01	0.04	0.01	0.00	0.05	0.02	0.00	0.06
	SMG	404	-48	-36	44	3.6	0.04	0.02	0.06	0.02	0.01	0.04	0.01	0.00	0.03	0.02	0.00	0.07	0.00	0.00	0.01
	PCC/Prec	1785	-8	-48	30	4.5	0.07	0.05	0.11	0.07	0.04	0.10	0.03	0.01	0.05	0.02	0.00	0.08	0.06	0.02	0.12
ReHo	AnG/LOC	529	-54	-58	24	4.9	0.01	0.00	0.02	0.03	0.01	0.05	0.02	0.01	0.04	0.00	0.00	0.02	0.00	0.00	0.00
	PCC	444	2	-58	26	4.3	0.04	0.02	0.06	0.03	0.01	0.05	0.01	0.00	0.03	0.02	0.00	0.08	0.01	0.00	0.05
**SEX by DIAGNOSIS**																					
VMHC*	sLOC	*138*	-30	-78	28	3.7	0.02	0.01	0.04	0.01	0.00	0.03	0.01	0.00	0.02	0.00	0.00	0.03	0.01	0.00	0.03

Abbreviations: PCC-iFC: posterior cingulate cortex intrinsic functional connectivity, VMHC: voxel-mirrored homotopic connectivity, ReHo: regional homogeneity, TOFC/OFG: temporal occiptal fusiform cortex/occiptal fusiform gyrus, sLOC: superior lateral occipital cortex, PCG/FP: paracingulate cortex/frontal pole, PCC/Prec: posterior cingulate gyrus/precuneus, CO/Ins: central operculum/insula, MFG: middle frontal gyrus, ACC: anterior cingulate cortex, SMG: supramarginal gyrus, AnG/LOC: angular gyrus/lateral occipital cortex. *Due to processing failure of two subjects for VMHC, the sample size comprised 1017 subjects instead of 1019 for ABIDE and 307 instead of 309 for EU-AIMS. Color-code green: findings meeting criteria for robustness and replicability (partial eta squared $\eta_p^2 \geq 0.01$); yellow: findings not meeting criteria for robustness and replicability.

3. Supplementary Figures

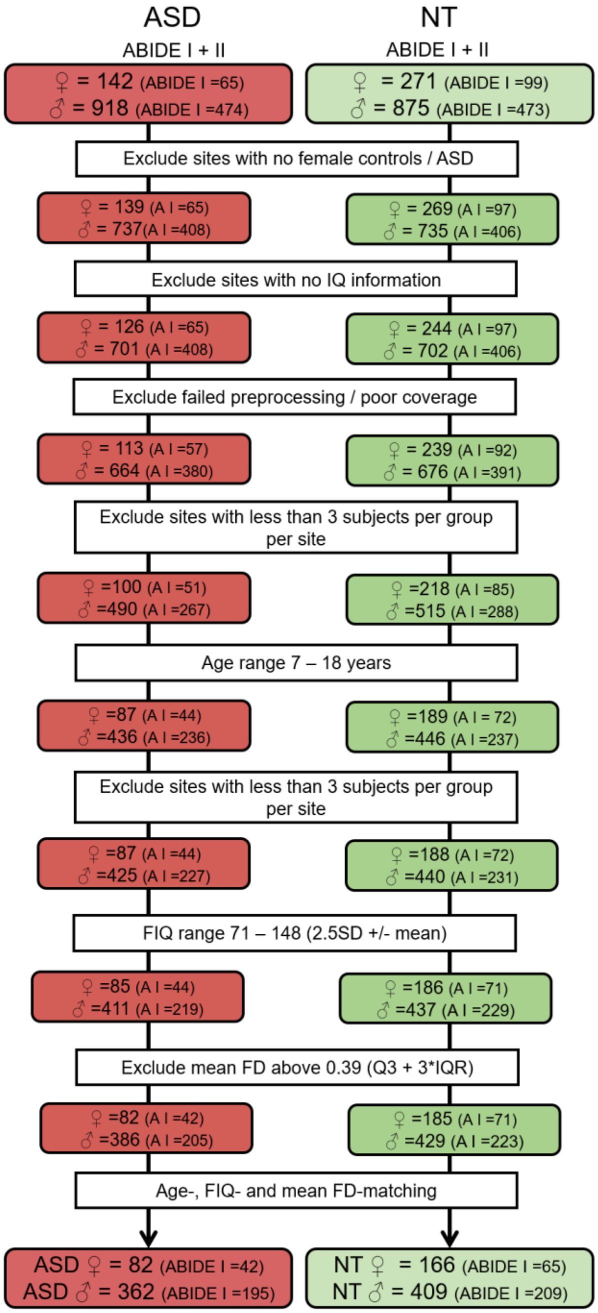

Fig S1. Selection flowchart for the ABIDE sample

The flowchart illustrates the selection process resulting in the final ABIDE I and II combined sample of 1019 subjects. At each flowchart step, the numbers outside the parentheses represent the total number of datasets across both ABIDE I and ABIDE II; in parenthesis are the number of datasets derived from ABIDE I (the resulting difference between these numbers would be the numbers for dataset stemming from ABIDE II). The rationale for each selection step is detailed in Supplementary Material. *Abbreviations*: ASD=autism spectrum disorder, NT=neurotypical, A I=ABIDE I.

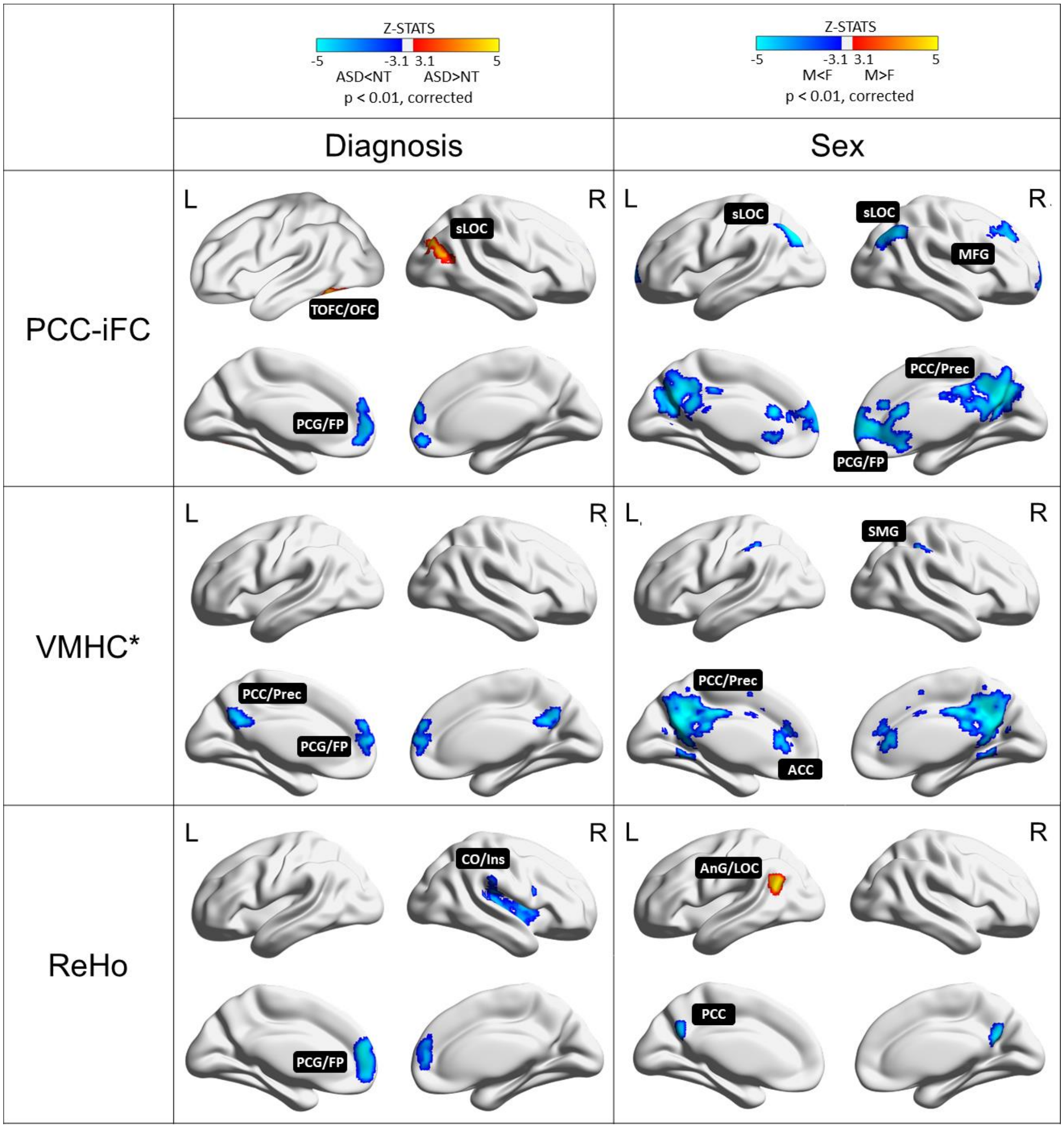

Fig S2. Main effects of diagnosis and sex in the ABIDE discovery sample

Significant results (Z>3.1, P<0.01, corrected) of voxel-wise discovery analyses conducted in the ABIDE dataset for main effects (ME) of diagnosis (left) and sex (right) for seed-based intrinsic functional connectivity of the posterior cingulate cortex (PCC), voxel-mirror homotopic connectivity (VMHC), and regional homogeneity (ReHo). Significant clusters are overlaid on inflated brain maps generated by BrainNet Viewer. No significant effects were detected for degree centrality or fractional amplitude of low frequency fluctuations. ME Diagnosis: PCC-iFC: bilateral paracingulate cortex and frontal pole (PCG/FP), superior lateral occipital cortex (sLOC), temporal occipital fusiform cortex and occipital fusiform gyrus (TOFC/OFC); VMHC: bilateral posterior cingulate gyrus and precuneus (PCC/Prec), PCG/FP; ReHo: PCG/FP, central operculum and insula (CO/Ins). ME Sex: PCC-iFC: bilateral sLOC, middle frontal gyrus (MFG), bilateral PCC/Prec, bilateral PCG/FP; VMHC: bilateral PCC/Prec, bilateral anterior cingulate cortex

(ACC); ReHo: bilateral PCC, angular gyrus and lateral occipital cortex (AnG/LOC). See Table S6 for details on each cluster sizes. *Due to processing failure of two subjects for VMHC, the sample size comprised 1017 subjects instead of 1019.

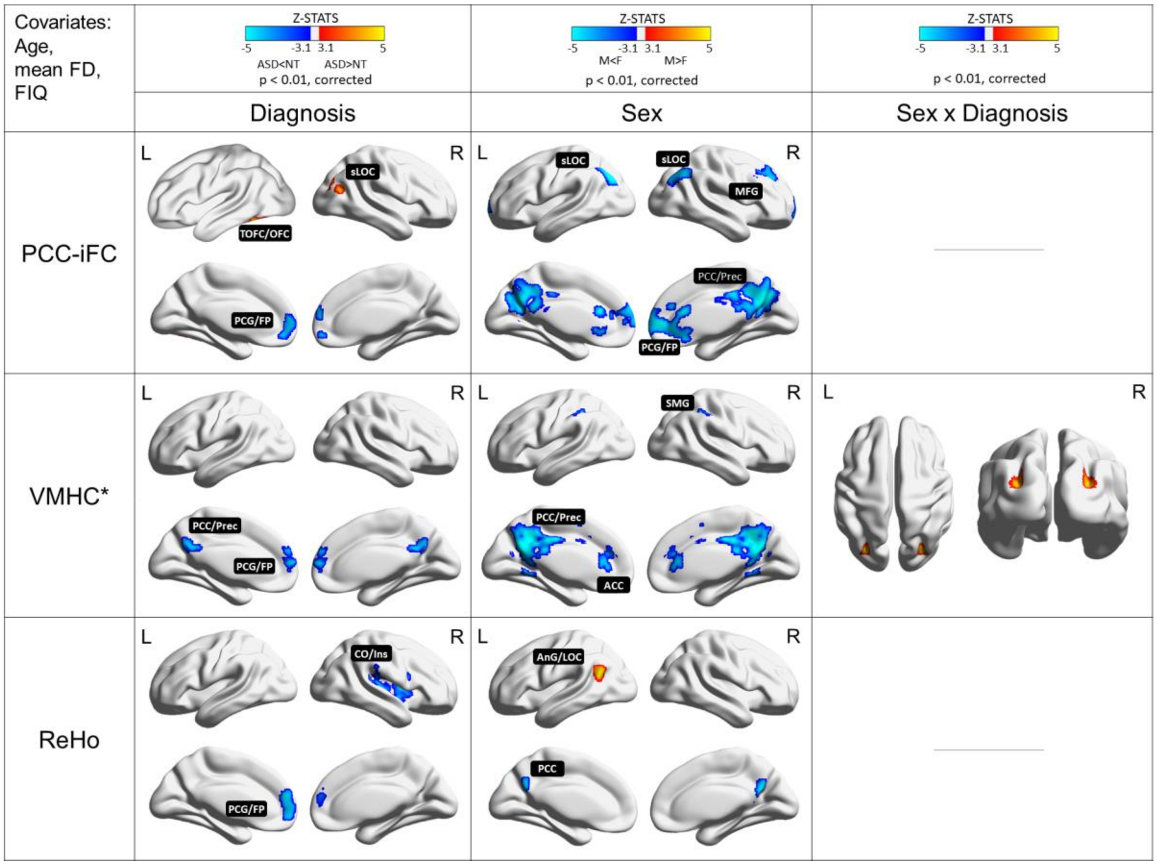

Fig S3. Main effects of diagnosis and sex in the ABIDE discovery sample when additionally covarying for FIQ

Including full-scale IQ (FIQ) as a nuisance regressor in addition to age and mean FD in the voxel-wise model yielded significant (Z>3.1, P<0.01, corrected) findings highly similar to those observed in discovery analyses across main effects (ME) of diagnosis (left) and sex (right), and their interaction. As in the discovery approach, analyses were conducted for seed-based intrinsic functional connectivity of the posterior cingulate cortex- (iFC-PCC), voxel-mirror homotopic connectivity (VMHC), and regional homogeneity (ReHo). Significant clusters are overlaid on inflated brain maps generated by BrainNet Viewer. No significant effects were detected for degree centrality or fractional amplitude of low frequency fluctuations. ME Diagnosis: PCC-iFC: bilateral paracingulate cortex and frontal pole (PCG/FP), superior lateral occipital cortex (sLOC), temporal occipital fusiform cortex and occipital fusiform gyrus (TOFC/OFC); VMHC: bilateral posterior cingulate gyrus and precuneus (PCC/Prec), PCG/FP; ReHo: PCG/FP, central operculum and insula (CO/Ins). ME Sex: PCC-iFC: bilateral sLOC, middle frontal gyrus (MFG), bilateral PCC/Prec, bilateral PCG/FP; VMHC: bilateral PCC/Prec, bilateral anterior cingulate cortex (ACC); ReHo: bilateral PCC, angular gyrus and lateral occipital cortex (AnG/LOC). Sex-by-diagnosis: VMHC: bilateral dorsolateral occipital cortex. *Due to processing failure of two subjects for VMHC, the sample size comprised 1017 subjects instead of 1019.

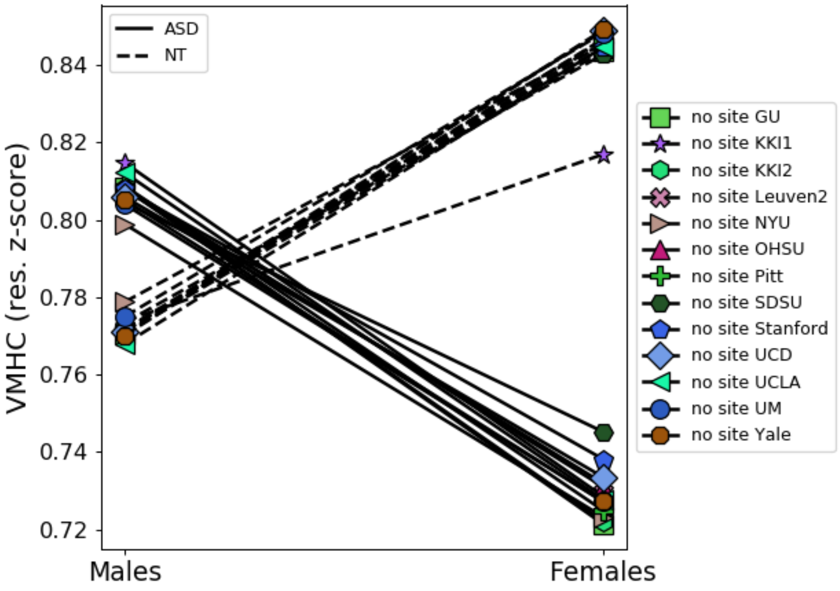

Fig S4. Stability of sex-by-diagnosis interaction effect

Inter-site stability of the sex-by-diagnosis interaction pattern was assessed after extracting group means at the mask corresponding to the clusters showing a significant interaction in the discovery analyses and then deriving the group mean when leaving one acquisition site out at the time. The pattern of results was unchanged. Different sites in ABIDE are color-coded in the legend on the right. Due to processing failure of two subjects for VMHC, the sample size comprised 1017 subjects. *Abbreviations*: ASD=autism spectrum disorder, NT=neurotypical, PCC-iFC=posterior cingulate cortex intrinsic functional connectivity (x=0, y=-53, z=26), VMHC=voxel-mirrored homotopic connectivity, ReHo=regional homogeneity, L=left, R=right. Different sites in ABIDE are color-coded on the top left.

References

1. Lord C, Risi S, Lambrecht L, Cook EH, Leventhal BL, DiLavore PC, et al. The autism diagnostic observation schedule-generic: a standard measure of social and communication deficits associated with the spectrum of autism. J Autism Dev Disord. 2000;30:205–23.

2. Lord C, Rutter M, Le Couteur A. Autism Diagnostic Interview-Revised: a revised version of a diagnostic interview for caregivers of individuals with possible pervasive developmental disorders. J Autism Dev Disord. 1994;24:659–85.

3. Jenkinson M, Bannister P, Brady M, Smith S. Improved optimization for the robust and accurate linear registration and motion correction of brain images. Neuroimage. 2002;17:825–41.

4. Hus V, Gotham K, Lord C. Standardizing ADOS domain scores: Separating severity of social affect and restricted and repetitive behaviors. J Autism Dev Disord. 2014;44:2400–12.

5. Gotham K, Pickles A, Lord C. Standardizing ADOS scores for a measure of severity in autism spectrum disorders. J Autism Dev Disord. 2009;39:693–705.

6. Loth E, Charman T, Mason L, Tillmann J, Jones EJH, Wooldridge C, et al. The EU-AIMS Longitudinal European Autism Project (LEAP): Design and methodologies to identify and validate stratification biomarkers for autism spectrum disorders. Mol Autism. 2017;8:24.

7. Irimia A, Torgerson CM, Jacokes ZJ, Van Horn JD. The connectomes of males and females with autism spectrum disorder have significantly different white matter connectivity densities. Sci Rep. 2017;7:46401.

8. Lawrence KE, Hernandez LM, Bowman HC, Padgaonkar NT, Fuster E, Jack A, et al. Sex Differences in Functional Connectivity of the Salience, Default Mode, and Central Executive Networks in Youth with ASD. Cereb Cortex. 2020;30:5107–5120.

9. Hernandez LM, Lawrence KE, Padgaonkar NT, Inada M, Hoekstra JN, Lowe JK, et al. Imaging-genetics of sex differences in ASD: distinct effects of OXTR variants on brain connectivity. Transl Psychiatry. 2020;10:82.

10. Carr T. Autism Diagnostic Observation Schedule. Encycl Autism Spectr Disord.

2013. p. 349–56.
11. Bruni TP. Test Review: Social Responsiveness Scale–Second Edition (SRS-2)ConstantinoJ. N.GruberC. P. (2012). Social Responsiveness Scale–Second Edition (SRS-2). Torrance, CA: Western Psychological Services. J Psychoeduc Assess. 2014;32.
12. Andrews-Hanna JR, Snyder AZ, Vincent JL, Lustig C, Head D, Raichle MEE, et al. Disruption of Large-Scale Brain Systems in Advanced Aging. Neuron. 2007;56:924–35.
13. Di Martino A, Yan CG, Li Q, Denio E, Castellanos FX, Alaerts K, et al. The autism brain imaging data exchange: Towards a large-scale evaluation of the intrinsic brain architecture in autism. Mol Psychiatry. 2014;19:659–67.
14. Floris DL, Lai MC, Nath T, Milham MP, Di Martino A. Network-specific sex differentiation of intrinsic brain function in males with autism. Mol Autism. 2018;9:17.
15. Alaerts K, Swinnen SP, Wenderoth N. Sex differences in autism: A resting-state fMRI investigation of functional brain connectivity in males and females. Soc Cogn Affect Neurosci. 2016;11:1002–16.
16. Assaf M, Jagannathan K, Calhoun VD, Miller L, Stevens MC, Sahl R, et al. Abnormal functional connectivity of default mode sub-networks in autism spectrum disorder patients. Neuroimage. 2010;53:247–56.
17. Lynch CJ, Uddin LQ, Supekar K, Khouzam A, Phillips J, Menon V. Default mode network in childhood autism: Posteromedial cortex heterogeneity and relationship with social deficits. Biol Psychiatry. 2013;74:212–9.
18. Lau WKW, Leung MK, Lau BWM. Resting-state abnormalities in Autism Spectrum Disorders: A meta-analysis. Sci Rep. 2019;9:3892.
19. Ypma RJF, Moseley RL, Holt RJ, Rughooputh N, Floris DL, Chura LR, et al. Default Mode Hypoconnectivity Underlies a Sex-Related Autism Spectrum. Biol Psychiatry Cogn Neurosci Neuroimaging. 2016;1:364–71.
20. Dumais KM, Chernyak S, Nickerson LD, Janes AC. Sex differences in default mode and dorsal attention network engagement. PLoS One. 2018;13:e0199049. https://doi.org/10.1371/journal.pone.0199049.
21. Scheinost D, Finn ES, Tokoglu F, Shen X, Papademetris X, Hampson M, et al. Sex differences in normal age trajectories of functional brain networks. Hum Brain Mapp. 2015;36:1524–35.

22. Biswal BB, Mennes M, Zuo XN, Gohel S, Kelly C, Smith SM, et al. Toward discovery science of human brain function. Proc Natl Acad Sci U S A. 2010;107:4734–9.
23. Zuo XN, Kelly C, Di Martino A, Mennes M, Margulies DS, Bangaru S, et al. Growing together and growing apart: Regional and sex differences in the lifespan developmental trajectories of functional homotopy. J Neurosci. 2010;30:15034–43.
24. Mazziotta JC, Toga AW, Evans A, Fox P, Lancaster J. A probabilistic atlas of the human brain: Theory and rationale for its development. Neuroimage. 1995;2:89–101.
25. Kozhemiako N, Vakorin V, Nunes AS, Iarocci G, Ribary U, Doesburg SM. Extreme male developmental trajectories of homotopic brain connectivity in autism. Hum Brain Mapp. 2019;40:987–1000.
26. Hahamy A, Behrmann M, Malach R. The idiosyncratic brain: Distortion of spontaneous connectivity patterns in autism spectrum disorder. Nat Neurosci. 2015;18:302–9.
27. King JB, Prigge MBD, King CK, Morgan J, Weathersby F, Fox JC, et al. Generalizability and reproducibility of functional connectivity in autism. Mol Autism. 2019;10:27.
28. Dinstein I, Pierce K, Eyler L, Solso S, Malach R, Behrmann M, et al. Disrupted Neural Synchronization in Toddlers with Autism. Neuron. 2011;70:1218–25.
29. Zang Y, Jiang T, Lu Y, He Y, Tian L. Regional homogeneity approach to fMRI data analysis. Neuroimage. 2004;22:394–400.
30. Bevan JM, Kendall MG. Rank Correlation Methods. Stat. 1971;20:74.
31. Kozhemiako N, Nunes AS, Vakorin V, Iarocci G, Ribary U, Doesburg SM. Alterations in Local Connectivity and Their Developmental Trajectories in Autism Spectrum Disorder: Does Being Female Matter? Cereb Cortex. 2020;30:5166–79.
32. Paakki JJ, Rahko J, Long X, Moilanen I, Tervonen O, Nikkinen J, et al. Alterations in regional homogeneity of resting-state brain activity in autism spectrum disorders. Brain Res. 2010;1321:169–79.
33. Keown CL, Shih P, Nair A, Peterson N, Mulvey ME, Müller RA. Local functional overconnectivity in posterior brain regions is associated with symptom severity in autism spectrum disorders. Cell Rep. 2013;5:567–72.
34. Maximo JO, Keown CL, Nair A, Müller RA. Approaches to local connectivity in

autism using resting state functional connectivity MRI. Front Hum Neurosci. 2013;7:605.
35. Nair S, Jao Keehn RJ, Berkebile MM, Maximo JO, Witkowska N, Müller RA. Local resting state functional connectivity in autism: site and cohort variability and the effect of eye status. Brain Imaging Behav. 2018;12:168–79.
36. Shukla DK, Keehn B, Müller RA. Regional homogeneity of fMRI time series in autism spectrum disorders. Neurosci Lett. 2010;476:46–51.
37. Takeuchi H, Taki Y, Nouchi R, Yokoyama R, Kotozaki Y, Nakagawa S, et al. Regional homogeneity, resting-state functional connectivity and amplitude of low frequency fluctuation associated with creativity measured by divergent thinking in a sex-specific manner. Neuroimage. 2017;152:258–69.
38. Xu C, Li C, Wu H, Wu Y, Hu S, Zhu Y, et al. Gender differences in cerebral regional homogeneity of adult healthy volunteers: A resting-state fMRI study. Biomed Res Int. 2015;2015:183074.
39. Dai XJ, Gong HH, Wang YX, Zhou FQ, Min YJ, Zhao F, et al. Gender differences in brain regional homogeneity of healthy subjects after normal sleep and after sleep deprivation: A resting-state fMRI study. Sleep Med. 2012;13:720–7.
40. Zuo XN, Ehmke R, Mennes M, Imperati D, Castellanos FX, Sporns O, et al. Network centrality in the human functional connectome. Cereb Cortex. 2012;22:1862–75.
41. Yan CG, Craddock RC, Zuo XN, Zang YF, Milham MP. Standardizing the intrinsic brain: Towards robust measurement of inter-individual variation in 1000 functional connectomes. Neuroimage. 2013;80:246–62.
42. Holiga Š, Hipp JF, Chatham CH, Garces P, Spooren W, D'Ardhuy XL, et al. Patients with autism spectrum disorders display reproducible functional connectivity alterations. Sci Transl Med. 2019;11:eaat9223. https://doi.org/10.1126/scitranslmed.aat9223.
43. Wan B, Wang Z, Jung M, Lu Y, He H, Chen Q, et al. Effects of the Co-occurrence of Anxiety and Attention-Deficit/Hyperactivity Disorder on Intrinsic Functional Network Centrality among Children with Autism Spectrum Disorder. Autism Res. 2019;12:1057–68.
44. Di Martino A, Zuo XN, Kelly C, Grzadzinski R, Mennes M, Schvarcz A, et al. Shared and distinct intrinsic functional network centrality in autism and attention-deficit/hyperactivity disorder. Biol Psychiatry. 2013;74:623–32.

45. Itahashi T, Yamada T, Watanabe H, Nakamura M, Ohta H, Kanai C, et al. Alterations of local spontaneous brain activity and connectivity in adults with high-functioning autism spectrum disorder. Mol Autism. 2015;6:30.
46. Zou QH, Zhu CZ, Yang Y, Zuo XN, Long XY, Cao QJ, et al. An improved approach to detection of amplitude of low-frequency fluctuation (ALFF) for resting-state fMRI: Fractional ALFF. J Neurosci Methods. 2008;172:137–41.
47. Pretzsch CM, Voinescu B, Mendez MA, Wichers R, Ajram L, Ivin G, et al. The effect of cannabidiol (CBD) on low-frequency activity and functional connectivity in the brain of adults with and without autism spectrum disorder (ASD). J Psychopharmacol. 2019;33:1141–8.
48. Jung M, Tu Y, Lang CA, Ortiz A, Park J, Jorgenson K, et al. Decreased structural connectivity and resting-state brain activity in the lateral occipital cortex is associated with social communication deficits in boys with autism spectrum disorder. Neuroimage. 2019;190:205–12.
49. Smith SM. Fast robust automated brain extraction. Hum Brain Mapp. 2002;17:143–55.
50. Zhang Y, Brady M, Smith S. Segmentation of brain MR images through a hidden Markov random field model and the expectation-maximization algorithm. IEEE Trans Med Imaging. 2001;20:45–57.
51. Avants BB, Tustison NJ, Wu J, Cook PA, Gee JC. An open source multivariate framework for N-tissue segmentation with evaluation on public data. Neuroinformatics. 2011;9:381–400.
52. Behzadi Y, Restom K, Liau J, Liu TT. A component based noise correction method (CompCor) for BOLD and perfusion based fMRI. Neuroimage. 2007;37:90–101.
53. Pruim RHR, Mennes M, van Rooij D, Llera A, Buitelaar JK, Beckmann CF. ICA-AROMA: A robust ICA-based strategy for removing motion artifacts from fMRI data. Neuroimage. 2015;112:267–77.
54. Greve DN, Fischl B. Accurate and robust brain image alignment using boundary-based registration. Neuroimage. 2009;48:63–72.
55. Johnson WE, Li C, Rabinovic A. Adjusting batch effects in microarray expression data using empirical Bayes methods. Biostatistics. 2007;8:118–27.

56. Fortin JP, Parker D, Tunç B, Watanabe T, Elliott MA, Ruparel K, et al. Harmonization of multi-site diffusion tensor imaging data. Neuroimage. 2017;161:149–70.
57. Fortin JP, Cullen N, Sheline YI, Taylor WD, Aselcioglu I, Cook PA, et al. Harmonization of cortical thickness measurements across scanners and sites. Neuroimage. 2018;167:104–20.
58. Yu M, Linn KA, Cook PA, Phillips ML, McInnis M, Fava M, et al. Statistical harmonization corrects site effects in functional connectivity measurements from multi-site fMRI data. Hum Brain Mapp. 2018;39:4213–27.
59. Fox MD, Snyder AZ, Vincent JL, Corbetta M, Van Essen DC, Raichle ME. The human brain is intrinsically organized into dynamic, anticorrelated functional networks. Proc Natl Acad Sci U S A. 2005;102:9673–8.
60. Liu TT, Nalci A, Falahpour M. The global signal in fMRI: Nuisance or Information? Neuroimage. 2017;150:213–29.
61. Beckmann CF, Smith SM. Probabilistic Independent Component Analysis for Functional Magnetic Resonance Imaging. IEEE Trans Med Imaging. 2004;23:137–52.
62. Birn RM. The role of physiological noise in resting-state functional connectivity. Neuroimage. 2012;62:864–70.
63. Li J, Kong R, Liégeois R, Orban C, Tan Y, Sun N, et al. Global signal regression strengthens association between resting-state functional connectivity and behavior. Neuroimage. 2019;196:126–41.
64. Fox MD, Zhang D, Snyder AZ, Raichle ME. The global signal and observed anticorrelated resting state brain networks. J Neurophysiol. 2009;101:3270–83.
65. Hus V, Lord C. The autism diagnostic observation schedule, module 4: Revised algorithm and standardized severity scores. J Autism Dev Disord. 2014;44:1996–2012.
