# Supplementary material for: Towards robust and replicable sex differences in the intrinsic brain function of autism
Source: Mol Autism. 2021 Mar 1;12:19. doi: 10.1186/s13229-021-00415-z (PMC7923310; doi:10.1186/s13229-021-00415-z)
Supplement: Supplementary file 2 — Additional file 2. Stability of main effects of diagnosis and sex. [file 13229_2021_415_MOESM2_ESM.pdf]

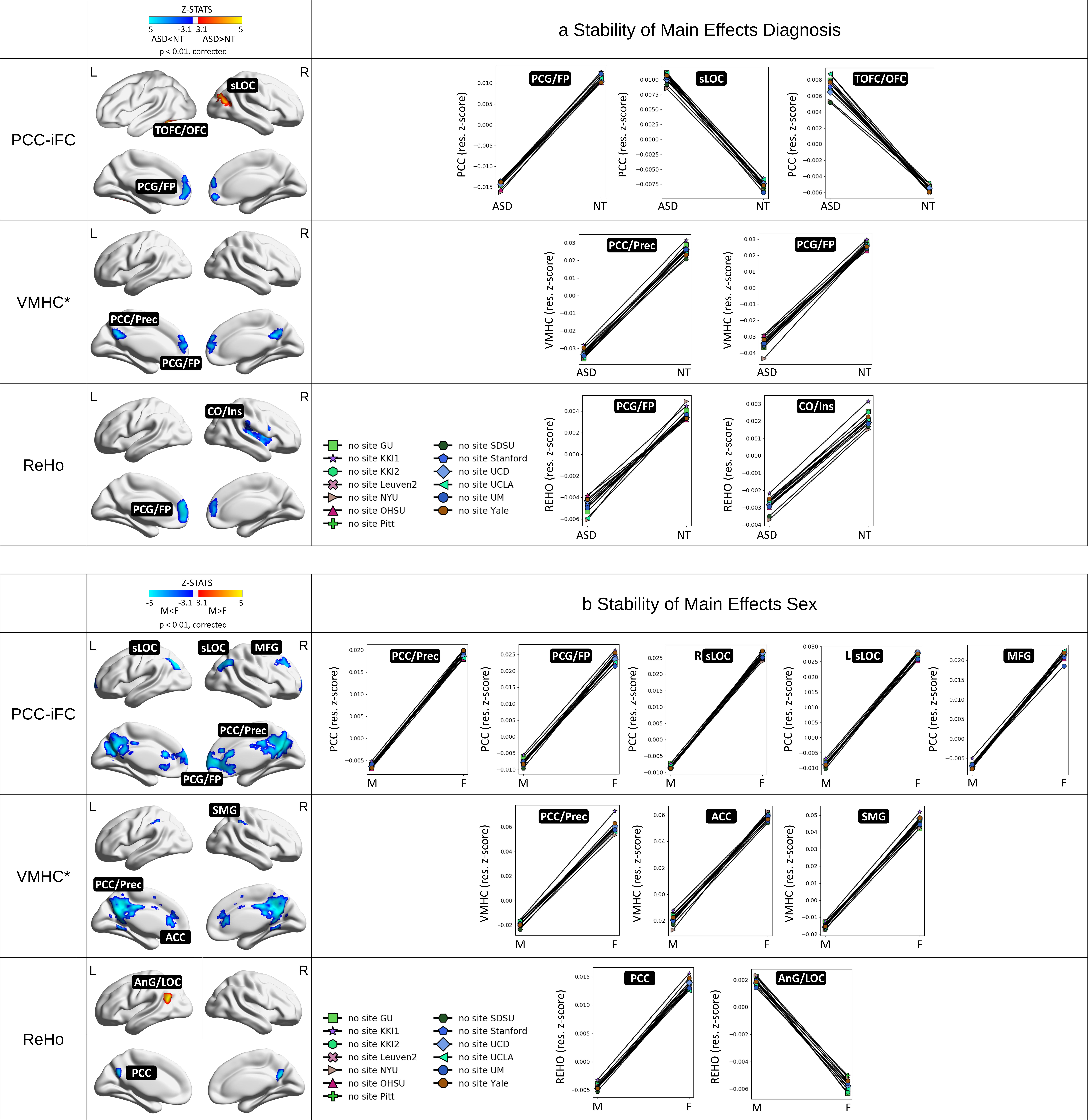

Z-STATS
-5 -3.1 3.1 5
ASD<NT ASD>NT
p < 0.01, corrected
a Stability of Main Effects Diagnosis
PCC-iFC
VMHC*
ReHo
L
R
sLOC
TOFC/OFC
PCG/FP
PCC/Prec
CO/Ins
PCC (res. z-score)
VMHC (res. z-score)
REHO (res. z-score)
ASD
NT
no site GU
no site KKI1
no site KKI2
no site Leuven2
no site NYU
no site OHSU
no site Pitt
no site SDSU
no site Stanford
no site UCD
no site UCLA
no site UM
no site Yale
Z-STATS
-5 -3.1 3.1 5
M<F M>F
p < 0.01, corrected
b Stability of Main Effects Sex
PCC-iFC
VMHC*
ReHo
sLOC
MFG
PCC/Prec
PCG/FP
R sLOC
L sLOC
SMG
ACC
AnG/LOC
PCC
M
F

Fig S5. Stability of main effects

Inter-site stability was assessed after extracting group means at masks corresponding to the clusters showing significant main effects of diagnosis (a) and sex (b) in the discovery analyses and then deriving the group mean when leaving one acquisition site out at the time. The pattern of results was unchanged. Different ABIDE sites are color-coded on legend on the side. Due to processing failure of two subjects for VMHC, the sample size comprised 1017 subjects. *Abbreviations*: ASD=autism spectrum disorder, NT=neurotypical, PCC-iFC=posterior cingulate cortex intrinsic functional connectivity (x=0, y=-53, z=26), VMHC=voxel-mirrored homotopic connectivity, ReHo=regional homogeneity, L=left, R=right. Different sites in ABIDE are color-coded on the top left. Data are shown as residuals obtained after regressing out mean framewise displacement and age effects.
