# Supplementary material for: Towards robust and replicable sex differences in the intrinsic brain function of autism
Source: Mol Autism. 2021 Mar 1;12:19. doi: 10.1186/s13229-021-00415-z (PMC7923310; doi:10.1186/s13229-021-00415-z)
Supplement: Supplementary file 3 — Additional file 3. Robustness and replicability of main effects of diagnosis and sex. [file 13229_2021_415_MOESM3_ESM.pdf]

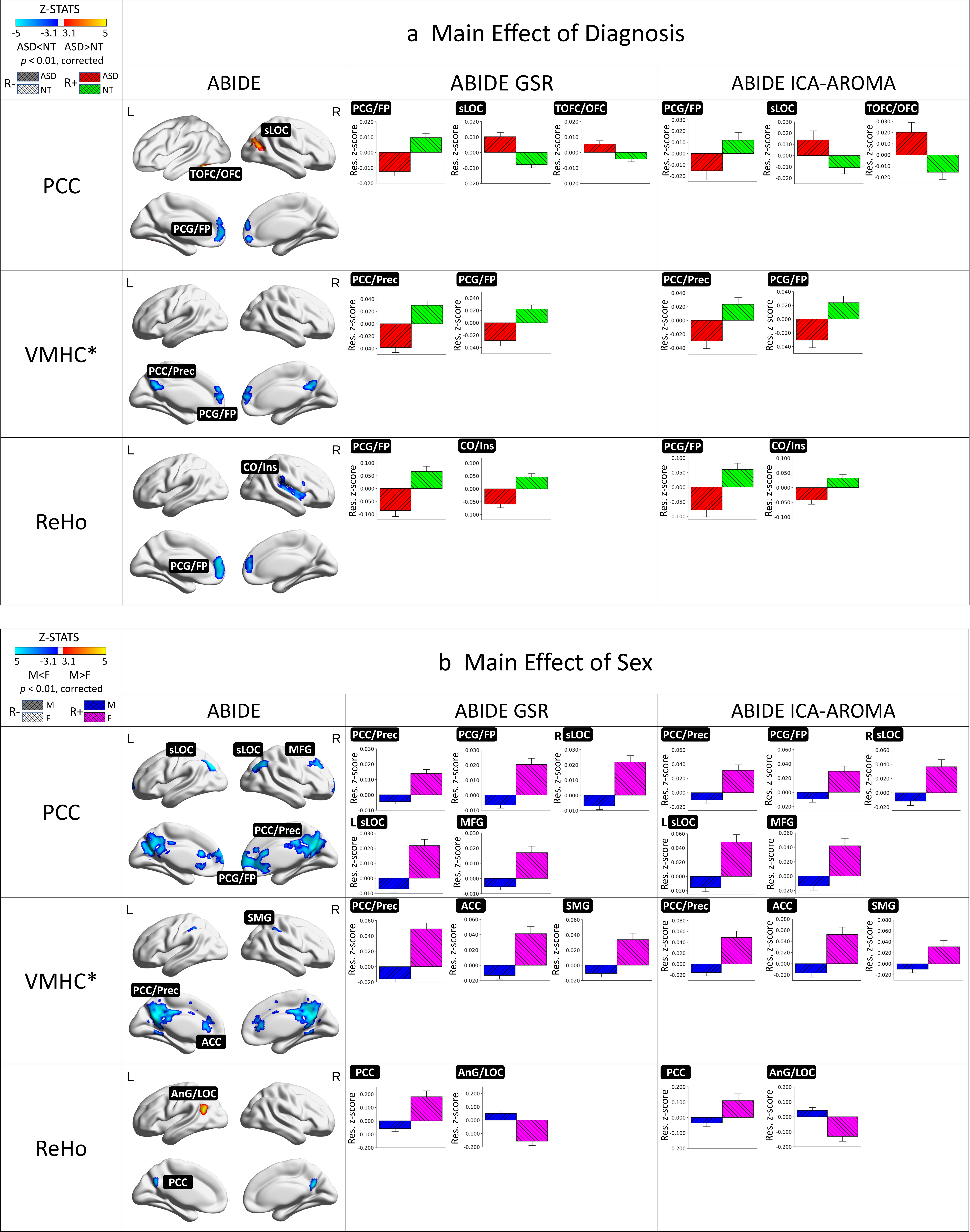

Z-STATS
-5 -3.1 3.1 5
ASD<NT ASD>NT
p < 0.01, corrected
R- ASD NT R+ ASD NT
a Main Effect of Diagnosis
ABIDE
ABIDE GSR
ABIDE ICA-AROMA
PCC
VMHC*
ReHo
L R
sLOC
TOFC/OFC
PCG/FP
PCC/Prec
CO/Ins
Res. z-score
Z-STATS
-5 -3.1 3.1 5
M<F M>F
p < 0.01, corrected
R- M F R+ M F
b Main Effect of Sex
ABIDE
ABIDE GSR
ABIDE ICA-AROMA
PCC
VMHC*
ReHo
L R
sLOC
MFG
PCC/Prec
PCG/FP
R sLOC
L sLOC
SMG
ACC
AnG/LOC
Res. z-score

Fig S6. Robustness to nuisance corrections of main effects of diagnosis and sex

Cluster-level replication of the results emerging from the voxel-wise discovery analyses in the ABIDE dataset preprocessed using CompCor for the statistically significant main effects of diagnosis (a) and sex (b) after preprocessing with GSR and with ICA-AROMA. The second column on the left shows the clusters (Z>3.1, P<0.01, corrected) with significant diagnostic and sex effects for posterior cingulate cortex intrinsic functional connectivity (PCC-iFC), voxel-mirrored homotopic connectivity (VMHC), and regional homogeneity (ReHo). Results are overlaid on inflated brain maps generated by BrainNet Viewer. The bar plots represent the residual means resulting from regressing out diagnosis or sex effects depending on the desired main effect from the cluster means. a) The ABIDE GSR and ABIDE ICA-AROMA columns illustrate, for each of these R-fMRI indices, the diagnostic group mean pattern across clusters with a diagnostic effect size of $\eta_p^2 \geq 0.01$. Color codes: Red=ASD; Green=NT. PCC-iFC: bilateral paracingulate cortex and frontal pole (PCG/FP), superior lateral occipital cortex (sLOC), temporal occipital fusiform cortex and occipital fusiform gyrus (TOFC/OFC); VMHC: bilateral posterior cingulate gyrus and precuneus (PCC/Prec), PCG/FP; ReHo: PCG/FP, central operculum and insula (CO/Ins). ABIDE GSR: 7 out of 7 main effects of diagnosis replicated (100%). ABIDE ICA-AROMA: 7 out of 7 main effects of diagnosis replicated (100%). b) The ABIDE GSR and ABIDE ICA-AROMA columns illustrate, for each of these R-fMRI indices, the sex group mean pattern across clusters with an effect size of $\eta_p^2 \geq 0.01$. Color codes: Blue=males; Pink=females. PCC-iFC: bilateral sLOC, middle frontal gyrus (MFG), bilateral PCC/Prec, bilateral PCG/FP; VMHC: bilateral PCC/Prec, bilateral anterior cingulate cortex (ACC); ReHo: bilateral PCC, angular gyrus and lateral occipital cortex (AnG/LOC). ABIDE GSR: 10 out of 10 main effects of diagnosis replicated (100%). ABIDE ICA-AROMA: 10 out of 10 main effects of diagnosis replicated (100%). Due to processing failure of two subjects for VMHC, the sample size comprised 1017 subjects instead of 1019. VMHC data are shown as residuals obtained after regressing out mean framewise displacement and age effects. *Abbreviations:* ASD=autism spectrum disorder, NT=neurotypical, M=males, F=females, CompCor=component base noise reduction, GSR=Global Signal Regression, ICA-AROMA=independent component analysis – automatic removal of motion artifacts, PCC-iFC=posterior cingulate cortex intrinsic functional connectivity (x=0, y=-53, z=26), VMHC=voxel-mirrored homotopic connectivity, ReHo=regional homogeneity, L=left, R=right, R+=replication based on same direction of results and $\eta_p^2 \geq 0.01$, R-=non-replication of results (displayed in gray plots).

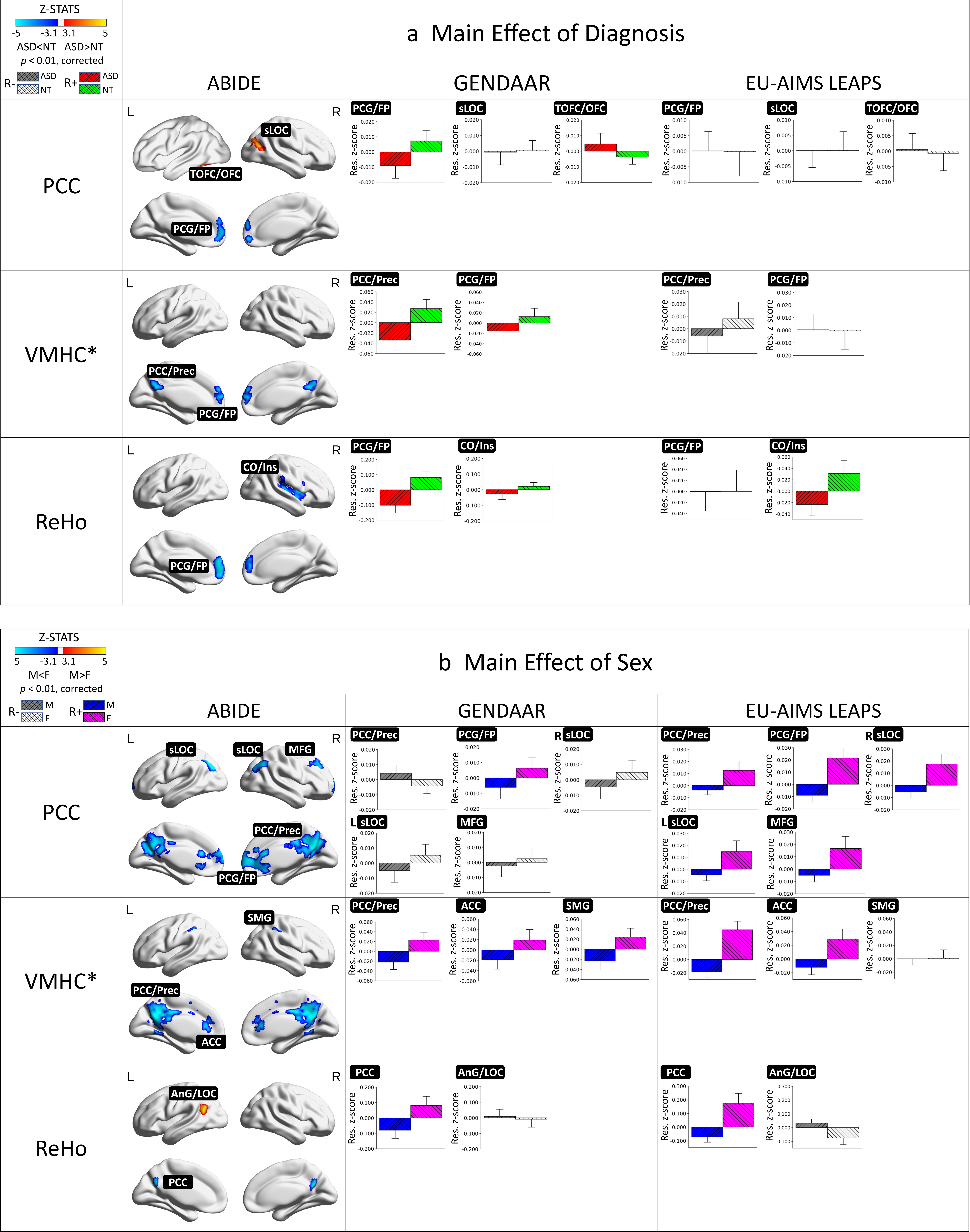
Z-STATS
-5 -3.1 3.1 5
ASD<NT ASD>NT
p < 0.01, corrected
R- ASD NT R+ ASD NT
a Main Effect of Diagnosis
ABIDE
GENDAAR
EU-AIMS LEAPS
PCC
VMHC*
ReHo
L R
sLOC
TOFC/OFC
PCG/FP
PCC/Prec
CO/Ins
Res. z-score
Z-STATS
-5 -3.1 3.1 5
M<F M>F
p < 0.01, corrected
R- M F R+ M F
b Main Effect of Sex
ABIDE
GENDAAR
EU-AIMS LEAPS
PCC
VMHC*
ReHo
sLOC
MFG
PCC/Prec
PCG/FP
R sLOC
L sLOC
SMG
ACC
AnG/LOC
PCC
Res. z-score

Fig S7. Replicability of main effects of diagnosis and sex

Cluster-level replication of the results emerging from the voxel-wise analyses in the ABIDE dataset for main effects of diagnosis (a) and sex (b) in the Gender Explorations of Neurogenetics and Development to Advance Autism Research (GENDAAR) and the EU-AIMS Longitudinal European Autism Project (LEAP) samples. The ABIDE column on the left shows the clusters ($Z>3.1$, $P<0.01$, corrected) with significant diagnostic and sex effects for posterior cingulate cortex intrinsic functional connectivity (PCC-iFC), voxel-mirrored homotopic connectivity (VMHC), and regional homogeneity (ReHo). No significant effects were detected for degree centrality and fractional amplitude of low frequency fluctuations. Results are overlaid on inflated brain maps generated by BrainNet Viewer. The bar plots represent the residual means resulting from the linear Gaussian regression for each group. a) The GENDAAR and EU-AIMS LEAP columns illustrate, for each R-fMRI index, the diagnostic group mean pattern across clusters with a diagnostic effect size of $\eta_p^2 \geq 0.01$. Color codes: Red=ASD; Green=NT. PCC-iFC: bilateral paracingulate cortex and frontal pole (PCG/FP), superior lateral occipital cortex (sLOC), temporal occipital fusiform cortex and occipital fusiform gyrus (TOFC/OFC); VMHC: bilateral posterior cingulate gyrus and precuneus (PCC/Prec), PCG/FP; ReHo: PCG/FP, central operculum and insula (CO/Ins). GENDAAR: 6 out of 7 main effects of diagnosis replicated (86%); EU-AIMS LEAP: 2 out of 7 main effects of diagnosis replicated (29%). b) The GENDAAR and EU-AIMS LEAP columns illustrate, for each of these R-fMRI index, the sex group mean pattern across clusters with an effect size of $\eta_p^2 \geq 0.01$. Color codes: Blue=males, Pink=females. PCC-iFC: bilateral sLOC, middle frontal gyrus (MFG), bilateral PCC/Prec, bilateral PCG/FP; VMHC: bilateral PCC/Prec, bilateral anterior cingulate cortex (ACC); ReHo: bilateral PCC, angular gyrus and lateral occipital cortex (AnG/LOC). GENDAAR: 5 out of 10 main effects of sex replicated (50%); EU-AIMS LEAP: 8 out of 10 main effects of sex replicated (80%). *Due to processing failure of two subjects for VMHC, the sample size comprised 1017 subjects instead of 1019. *Abbreviations:* ASD=autism spectrum disorder, NT=neurotypical, M=males, F=females, PCC-iFC=posterior cingulate cortex intrinsic functional connectivity (x=0, y=-53, z=26), VMHC=voxel-mirrored homotopic connectivity, ReHo=regional homogeneity, L=left, R=right, R+=replication based on same direction of results and $\eta_p^2 \geq 0.01$, R-=non-replication of results.
